# Quality of life and health status of hospitalized adults with congenital heart disease in Vietnam: a cross-sectional study

**DOI:** 10.1186/s12872-021-02026-1

**Published:** 2021-05-05

**Authors:** Thanh-Huong Truong, Ngoc-Thanh Kim, Mai-Ngoc Thi Nguyen, Doan-Loi Do, Hong Thi Nguyen, Thanh-Tung Le, Hong-An Le

**Affiliations:** 1 Vietnam National Heart Institute, Bach Mai Hospital, 78 Giai Phong Road, Dong Da District, Hanoi, 100000 Vietnam; 2 Department of Cardiology, Hanoi Medical University, 1 Ton That Tung Street, Dong Da District, Hanoi, 100000 Vietnam; 3 Thanh Nhan Hospital, 42 Thanh Nhan Street, Hai Ba Trung District, Hanoi, 100000 Vietnam; 4 School of Medicine and Pharmacy, Vietnam National University, 144 Xuan Thuy Road, Cau Giay District, Hanoi, 100000 Vietnam

**Keywords:** Quality of life, Health status, Adults with congenital heart disease, Hospitalized, Vietnam

## Abstract

**Background:**

Little is known about the quality of life (QOL) and health status of adults with congenital heart disease (CHD) in developing countries. Therefore, this study aimed to describe the QOL and health status of hospitalized adults with CHD in Vietnam and investigate the association between QOL and their biological-social characteristics.

**Methods:**

A cross-sectional study was conducted with 109 adults with CHD, hospitalized in the Vietnam National Heart Institute, between June and December 2019. Validated instruments to assess QOL and health status describing patient-reported outcomes were used, including the EuroQOL-5 Dimensions-5 Level, Satisfaction with Life Scale, and Hospital Anxiety and Depression Scale.

**Results:**

The mean scores on the EuroQOL-descriptive system (EQ-DS) and EuroQOL visual analogue scale (EQ-VAS) were 0.792 (SD = 0.122, 95% confidence interval [CI] 0.769–0.815) and 66.3 (SD = 12.5, 95% CI 63.9–68.7), respectively. A total of 9.2% (n = 9) patients experienced life dissatisfaction. The prevalence of anxiety and depression were 18.7% (n = 20) and 11% (n = 12), respectively. Scores of QOL in patients aged > 30 years were lower than in those aged ≤ 30 years. Stratified multivariate logistic regression revealed that poor QOL related to being unemployed/unstable employment (OR 4.43, 95% CI 1.71–11.47, *p* = 0.002), life dissatisfaction associated with unmarried status (OR 4.63, 95% CI 1.2–17.86, *p* = 0.026), anxiety regarding unemployment/unstable employment (OR 3.88, 95% CI 1.27–11.84, *p* = 0.017) and complex CHD/PAH (OR 4.84, 95% CI 1.33–17.54, *p* = 0.016), and depression regarding unemployment/unstable employment (OR 4.63, 95% CI 1.22–17.59, *p* = 0.003).

**Conclusions:**

Reduced QOL and elevated psychological problems were common experiences among hospitalized adults with CHD in Vietnam. Biological-social characteristics such as unmarried status, unemployment/unstable employment, and complex CHD/PAH related to poor QOL, life dissatisfaction, anxiety, and depression.

**Supplementary Information:**

The online version contains supplementary material available at 10.1186/s12872-021-02026-1.

## Background

Congenital heart disease (CHD) is a common cardiac structural abnormality that affects one in 100 live births, globally. Many countries have made efforts towards improving the diagnostic quality and treatment of CHD, which has resulted in many children with CHD surviving well into adulthood [1–3]. However, several adults with CHD continually face physical and psychosocial difficulties and experience declined quality of life (QOL) [4–7]. Therefore, QOL has become an important endpoint in the quality assessment of medical care in many countries. A recent study recognized the association between increased social activities and better QOL [8]. Thereby, current guidelines on adults with CHD addressed the comprehensive care provided by allied health professionals to improve physical-mental health and QOL [9]. In this context, knowledge of QOL and health status in adults with CHD is an emerging domain and deserves investigation. However, in the long term, many studies on QOL related to CHD have focused on children, adolescents, or their parents, despite the existence of a large gap in the knowledge regarding QOL and health status in adults with CHD, especially in developing countries [4, 10–15].

The discrepancy between the QOL status of adults with CHD in developed countries and developing countries is concerning. Previous studies conducted in developed countries showed high QOL in adults with CHD [4, 16, 17]. In contrast, in developing countries, the QOL of adults with CHD seems poorer than that of the healthier counterparts [15]. Notably, a high QOL is the result of good physical health, good psychological state, and positive social relationships. These items depend on medical intervention, healthcare systems, financial conditions of the concerned individual, the national economic conditions, and the sociocultural characteristics that differ between countries; for example, these characteristics may differ between Asian and European countries, low/middle-income and high-income countries, developing and developed countries. Recently, an international study found that the effect of country-specific characteristics of QOL score of adults with CHD differs by 10.5 points between countries with the highest and lowest QOL [10, 18]. From the abovementioned studies, we can conclude that researching QOL in adults with CHD in developing countries is essential. This would be the base for implementing important health and social policies to improve the QOL of adults with CHD in developing countries.

Vietnam is a densely populated, dynamic country in Southeast Asia, with a population of 97 million; it can be classified as a low-/middle-income country. Apart from economic development, the Vietnamese government has also focused on improving the healthcare system. In fact, a previous study has highlighted the screening programmes, diagnostic approaches, and treatment for patients with CHD throughout the country [19]. Owing to these programmes, many children have received timely interventions and have survived into adulthood. However, management with care is lacking for adults with CHD in Vietnam, including programmes for improving their QOL. In this context, the characteristics of Vietnamese patients may be generalised to adults with CHD in developing countries. Specifically, these characteristics include low level of education, unmarried status, unemployment, and unrepaired defects. However, we lack information about the assessed QOL in adults with CHD and its relationship with different sociodemographic characteristics in these countries. Therefore, the present study aimed to describe the QOL and health status of hospitalized adults with CHD in Vietnam and investigate the association between the QOL and the biological and social characteristics of these patients.

## Methods

### Study design

We performed a cross-sectional study at the Vietnam National Heart Institute, Bach Mai Hospital (Hanoi, Vietnam)—the largest hospital for adults with CHD in North Vietnam and the national referral cardiovascular hospital—between June and December 2019. Owing to the lack of availability of specialized outpatient services for adults with CHD, we recruited inpatients who were admitted for cardiac imaging, intervention, or surgery for CHD.

All patients included in this study provided informed consent. The protocol of the current study and ethics approval for the human subject study was obtained from the Science Boards and Ethics Committee of the Department of Cardiology, Hanoi Medical University (no: 6655/QD-DHYHN). The experiment protocol involving human subjects was adopted in accordance with the Declaration of Helsinki.

### Inclusion criteria

The inclusion criteria were (1) individuals with structural CHD confirmed by cardiac imaging and (2) aged ≥ 16 years. When physicians or cardiologists had patients with CHD, they would transfer these patients to our hospital to confirm defects wherein the adults with CHD were hospitalized for defects to be confirmed by specialists using cardiac imaging techniques. Transthoracic echocardiography was performed by experts on patients with CHD at the Vietnam National Heart Institute at admission. If structural CHD by transthoracic echocardiography was doubted, we confirmed structural CHD by transoesophageal echocardiography, cardiac computed tomography, and/or cardiac magnetic resonance imaging. After that, the specialist would explain the findings and provide treatment plans for each patient (intervention or surgery or only internal medicine).

In the study, we defined the age for reaching legal adulthood as 16 years, according to the current Law on Children of Vietnam (http://vbpl.vn/TW/Pages/vbpqen-toanvan.aspx?ItemID=11044). We also defined youth as the age cohort of 16–30 years, according to the current Youth Law of Vietnam (http://vanban.chinhphu.vn/portal/page/portal/chinhphu/hethongvanban?class_id=1&_page=1&mode=detail&document_id=200445).

### Exclusion criteria

Exclusion criteria for participants were the following: (1) known cardiac diseases other than CHD, (2) other known chronic diseases that require ongoing medical attention or limit activities of daily living, (3) having received post-operative care, (4) having acute medical conditions, (5) known neuropathy disorders and syndromes affecting cognitive abilities, and (6) emotional fragilities such as dissociative and conversion disorders (Codes F44 according to the International Statistical Classification of Diseases and Related Health Problems 10th Revision) identified by reviewing the participants’ medical records and during inpatient treatment. Notably, in case of a patient who was doubted to be emotionally fragile, a psychiatrist was consulted for final diagnosis.

### Sample size

The sample size was calculated using the following formula for cross-sectional studies: $N = \frac{{Z_{1 - \alpha /2}^{2} P\left( {1 - P} \right) }}{{d^{2} }}$, where N is the sample size, Z the statistic corresponding to the confidence level, P the expected prevalence, and d precision. However, we chose P from a recent international study with a prevalence rate of reduced QOL in adults with CHD of 29.7% [10] and a recent study in Germany with a prevalence rate of psychological problems in adults with CHD of 58.7% [20]. With a confidence interval (CI) of 95%, and a precision of 0.1, the sample size was calculated to be 94. To account for missing data, we added 15% to the sample size. Therefore, the final sample size was 108.

Additional file 1 shows the flow diagram of the selection strategy of adults with CHD, which provides information on the number of patients recruited and those determined ineligible for participation. From 290 adults with CHD, we excluded 181 for various reasons. Finally, we enrolled 109 adults with CHD in this study.

### Outcome measures

The patients completed a survey that included items of biological and social characteristics such as age, gender, marital status, employment status, educational level, CHD type, and CHD treatment. Subsequently, the following validated instruments describing patient-reported outcomes were completed to assess QOL and health status: EuroQOL-5 dimensions-5 level (EQ-5D-5L), Satisfaction with Life Scale (SWLS), and Hospital Anxiety and Depression Scale (HADS). Additionally, face-to-face interviews were conducted by one well-trained nurse. This nurse answered and clarified the questions and doubts of patients during the survey. She also ensured that patients completed the survey independently.

Primary outcomes measures were self-reported QOL, which was evaluated using EQ-5D-5L and SWLS, and self-reported psychosocial functioning that was evaluated by HADS. Secondary outcomes measures were socioeconomic status and the relationship between socioeconomic status and QOL and health status in adults with CHD.

### Vietnamese translation scales for QOL and health status

The EQ-5D-5L is a questionnaire used to assess health-related QOL and that includes EQ-descriptive system (EQ-DS) and EQ visual analogue scale (EQ-VAS); (Additional file 2, https://euroqol.org/eq-5d-instruments/sample-demo/). Here, we referred to the previous Vietnamese translation EQ-5D-5L version, which was developed by considering the health preferences of the general adult population of Vietnam and validated elsewhere [21]. The EQ-DS defines health based on five dimensions: Mobility, Self-Care, Usual Activities, Pain/Discomfort, and Anxiety/Depression. Responses are rated on a five-point Likert scale (no problems, slight problems, moderate problems, severe problems, and extreme problems). The value set for the EQ-DS was redesigned for the Vietnamese population on a scale ranging from 0 (worst imaginable QOL) to 1 (best imaginable QOL), while on the EQ-VAS, respondents rated the overall health on the day of the interview with scores ranging from 0 to 100, representing the worst and the best imaginable health state, respectively. Notably, poor QOL was defined as an EQ-VAS score of less than 65 [22].

The SWLS was a five-item instrument to measure general cognitive fundaments of life satisfaction. Each item is rated from one (strongly disagree) to seven (strongly agree) for a total score of 5–35 (Additional file 3, http://labs.psychology.illinois.edu/~ediener/SWLS.html**)**. A score of 20 represents a neutral point on the scale, while scores of 31–35, 26–30, 21–25, 15–19, 10–14, and 5–9 indicate that the respondent is extremely satisfied, satisfied, slightly satisfied, slightly dissatisfied, dissatisfied, and extremely dissatisfied with life, respectively [23]. In our study, we referred to the available Vietnamese translation version of the SWLS (https://eddiener.com/scales/7).

The HADS comprises 14 items (graded as 0–3), which include seven items each for symptoms of anxiety (HADS-Anxiety subscale, HADS-A) and depression (HADS-Depression subscale, HADS-D); Additional file 4, https://www.svri.org/sites/default/files/attachments/2016-01-13/HADS.pdf. The total scores for depression and anxiety range between 0 and 21. We considered a score of 8–10 to represent borderline abnormality and 11–21 to represent symptoms of anxiety or depression [24]. In this study, we referred to the Vietnamese translation version of the HADS, whose validity and reliability were confirmed in previous studies, with Cronbach's alpha of 0.80 for the HADS-D and 0.85 for the HADS-A [25, 26].

Following this, in our study, we modified the abovementioned Vietnamese translation scales (EQ-5D-5L, SWLS, and HADS). Some Vietnamese words were retranslated to make questions and options more understandable for interviewees. The process of modifying the Vietnamese translation scales included three stages. In stage one, the EQ-5D-5L, SWLS, and HADS were independently translated from English into Vietnamese by three professionals fluent in English: one cardiologist, one fifth-year medical student, and one translator without a medical background. In stage two, a medical expert compared all versions of the translations produced in the previous steps with the available translated version and agreed on the pre‐final version. Finally, in stage three, our research team, consisting of all the members conducting this study, discussed the pre‐final version, reached a consensus, and produced the final Vietnamese translation versions of the EQ-5D-5L, SWLS, and HADS (Additional file 5).

### Statistical analysis

Data were analysed by SPSS v22 (IBM Inc., Armonk, NY, USA). Normally distributed continuous variables were described as mean (standard deviation [SD], 95% CI) and non-normally distributed continuous variables were described as median and interquartile range. Nominal variables are presented as absolute numbers (*n*) and percentages. Frequencies and percentages were calculated for nominal variables. Differences in normally distributed continuous variables were assessed using the Student’s t-test, and differences in non-normally distributed variables were assessed using the Mann–Whitney U tests. Nominal variables between subgroups were compared using Chi-square tests or Fisher’s exact tests. Cohen's d was used to measure the effect size of independent samples t-test and Mann–Whitney U test. The univariable and multivariable forward logistic regression model using the forward stepwise method (likelihood ratio) was performed to evaluate the associations between the biological-social characteristics and poor QOL and the health status of the participants. In all analyses, a two-tailed *p* value below 0.05 was considered statistically significant.

## Results

### Patient characteristics

A total of 109 adults with CHD were enrolled in this study. The patients were predominantly women (n = 76, 69.7%). The participants’ overall mean age was 37.8 years (SD = 12.7, 95% CI 35.4–40.2); the mean age of men and women was 33.7 years (SD = 13.4, 95% CI 29.0–38.5) and 39.6 years (SD = 12.0, 95% CI 36.9–42.3), respectively. Most patients (n = 70, 64.2%) were older than 30 years. The characteristics of patients are summarized in Table 1. In addition, many patients had an education level of less than high school (n = 49, 45%), were unmarried (never married, widowed, divorced or separated; n = 30, 27.5%), were unemployed or had unstable employment (n = 27, 24.8%), and had complex CHD or pulmonary artery hypertension (PAH; n = 16, 14.7%). Notably, most patients had unrepaired CHD (n = 81, 74.3%) or repaired palliation (n = 5, 4.6%).

**Table 1:** Demographic characteristics of adults with congenital heart disease

Characteristics	N (%)
Gender	
Men	33 (30.3)
Women	76 (69.7)
Age groups	
16–30	39 (35.8)
31–40	31 (28.4)
41–50	18 (16.5)
51–60	14 (12.8)
61 +	7 (6.4)
Marital status	
Never married	23 (21.1)
Married/cohabiting	79 (72.5)
Window or divorce or separate	7 (6.4)
Educational level	
Secondary school and less	49 (45.0)
High school	19 (17.4)
Undergraduate and postgraduate	37 (33.9)
Missing data	4 (3.7)
Occupation	
Farmer	30 (27.5)
Blue-collar	22 (20.2)
White-collar	30 (27.5)
Student	6 (5.5)
Others	21 (19.3)
Employment status	
Stable	59 (54.1)
Homemaker or retired	14 (12.8)
Unstable	21 (19.3)
Unemployed	6 (5.5)
Student or other works	6 (5.5)
Missing data	3 (2.8)
Type of congenital heart disease	
Isolated atrial septal defect	53 (48.6)
Isolated ventricular septal defect	29 (26.6)
Isolated patent ductus arteriosus	19 (17.4)
Combined ventricular septal defect and patent ductus arteriosus	1 (0.9)
Combined atrial septal defect and pulmonary stenosis	1 (0.9)
Combined ventricular septal defect and pulmonary stenosis	2 (1.8)
Tetralogy of Fallot	3 (2.8)
Pulmonary atresia with ventricular septal defect	1 (0.9)
Congenital heart disease status	
Simple	93 (85.3)
Complex or pulmonary artery hypertension	16 (14.7)
Treatment for congenital heart disease	
Interventional/surgical correction	23 (21.1)
Interventional/surgical palliation	5 (4.6)
Unrepaired	81 (74.3)

### Characteristics of quality of life

The overall mean for EQ-DS and EQ-VAS was 0.792 (SD = 0.122, 95% CI 0.769–0.815) and 66.3 (SD = 12.5, 95% CI 63.9–68.7), respectively. The overall mean EQ-DS was significantly higher than EQ-VAS (*p* < 0.001, Student t-test). A significant number of adults with CHD had poor QOL, defined as EQ-VAS < 65 (n = 45, 41.3%). Table 2 summarises in detail the distribution of the EQ-5D-5L scale. Additionally, the most common problems measured by EQ-DS were pain/discomfort (n = 95, 87.2%), anxiety/depression (n = 83, 76.1%), mobility problems (n = 46, 42.2%), and problems with usual activities (n = 41, 37.6%), whereas the least reported complaint regarded self-care (n = 11, 10.1%).

**Table 2:** Profiles of EQ-5D-5L in adults with congenital heart disease

Scales		Score range									
		0–0.1	0.11–0.2	0.21–0.3	0.31–0.4	0.41–0.5	0.51–0.6	0.61–0.7	0.71–0.8	0.81–0.9	0.91–1
EuroQOL-Descriptive System	*N*	0	0	0	0	4	3	16	31	30	25
	%	0	0	0	0	3.7	2.8	14.7	28.4	27.5	22.9

		0–10	11–20	21–30	31–40	41–50	51–60	61–70	71–80	81–90	91–100
EuroQOL Visual Analogue Scale	*N*	0	1	0	4	13	27	36	25	2	1
	%	0	0.9	0	3.7	11.9	24.8	33	22.9	1.8	0.9

Domains of EuroQOL-Descriptive System		Levels				
		Extreme problems	Severe problems	Moderate problems	Slight problems	No problems
Mobility	*N*	0	0	6	40	63
	%	0	0	5.5	36.7	57.8
Self-care	*N*	0	0	0	11	98
	%	0	0	0	10.1	89.9
Usual activities	*N*	0	0	4	37	68
	%	0	0	3.7	33.9	62.4
Pain/discomfort	*N*	0	1	17	77	14
	%	0	0.9	15.6	70.6	12.8
Anxiety/depression	*N*	1	5	16	61	26
	%	0.9	4.6	14.7	56	23.9

### Characteristics of health status

The overall mean SWLS score was 25.2 ± 4.3 (95% CI 24.3–25.9). Specifically, 3.7% (n = 4) of patients reported being dissatisfied, 5.5% (n = 6) slightly dissatisfied, 2.8% (n = 3) neutral, 42.2% (n = 46) slightly satisfied, 36.7% (n = 40) satisfied, and 9.2% (n = 10) extremely satisfied. Regarding the HADS, the overall mean score for anxiety was 6.9 (SD = 4.4, 95% CI 6.1–7.8) and 5.9 (SD = 3.8, 95% CI 5.2–6.6) for depression. The anxiety component revealed that 18.7% (n = 20) of patients had experienced significant anxiety. Meanwhile, 11% (n = 12) of patients displayed significant symptoms of depression.

### Association between quality of life, health status, and biological-social characteristics in adults with congenital heart disease

As shown in Table 3, EQ-DS differs between subgroups. For EQ-DS, patients aged > 30 years had a lower score than those aged ≤ 30 years (0.764 [SD = 0.125], 95% CI 0.734–0.794 vs 0.841 [SD = 0.097], 95% CI 0.81–0.873, *p* = 0.001 overall; 0.766 [SD = 0.111], 95% CI 0.704–0.827 vs 0.853 [SD = 0.086], 95% CI 0.81–0.896, *p* = 0.016 in men; 0.764 [SD = 0.13], 95% CI 0.729–0.799 vs 0.831 [SD = 0.106], 95% CI 0.782–0.879, *p* = 0.039 in women; using the Student t-test; respectively), patients who had an education level lower than high school had a lower score than those with an education level of high school or higher (0.754 [SD = 0.13], 95% CI 0.716–0.791 vs 0.827 [SD = 0.104], 95% CI 0.8–0.855, *p* = 0.002 overall; 0.746 [SD = 0.132], 95% CI 0.652–0.841 vs 0.863 [SD = 0.061], 95% CI 0.835–0.891, *p* = 0.002 in men; using Student t-test; respectively), patients with an unemployed status/unstable employment had a lower score than those with an employed status (0.754 [SD = 0.136], 95% CI 0.7–0.808 vs 0.807 [SD = 0.114], 95% CI 0.782–0.833, *p* = 0.049 overall; 0.737 [SD = 0.151], 95% CI 0.598–0.877 vs 0.848 [SD = 0.071], 95% CI 0.817–0.878, *p* = 0.01 in men; using the Student t-test; respectively). Moreover, EQ-DS in employed women was lower than in employed men (0.79 [SD = 0.125], 95% CI 0.756–0.823 vs 0.848 [SD = 0.071], 95% CI 0.817–0.878, *p* = 0.037; using Student t-test; respectively).

**Table 3:** EQ-5D-5L by subgroups in adults with congenital heart disease

Groups	Total				Men				Women				*p* value*	Cohen’s d*
	Mean (SD)	95%CI	*p* value	Cohen’s d	Mean (SD)	95%CI	*p* value	Cohen’s d	Mean (SD)	95%CI	*p* value	Cohen’s d		
EuroQOL-Descriptive System (EQ-DS)														
Total	0.792 (0.121)	0.769–0.815	–	–	0.814 (0.106)	0.776–0.851	–	–	0.782 (0.127)	0.753–0.811	–	–	0.22	0.274
	(n = 109)				(n = 33)				(n = 76)					
Age groups														
16–30	0.841 (0.097)	0.81–0.873	0.001	0.688	0.853 (0.086)	0.81–0.896	0.016	0.901	0.831 (0.106)	0.782–0.879	0.039	0.565	0.472	0.228
	(n = 39)				(n = 18)				(n = 21)					
31+	0.764 (0.125)	0.734–0.794			0.766 (0.111)	0.704–0.827			0.764 (0.13)	0.729–0.799			0.964	0.017
	(n = 70)				(n = 15)				(n = 55)					
Marital status														
Married	0.795 (0.108)	0.771–0.819	0.659	0.091	0.795 (0.106)	0.739–0.851	0.339	0.338	0.795 (0.11)	0.767–0.823	0.056	0.49	0.999	0
	(n = 79)				(n = 16)				(n = 63)					
Unmarried	0.783 (0.152)	0.727–0.84			0.831 (0.107)	0.776–0.886			0.721 (0.183)	0.611–0.832			0.049	0.734
	(n = 30)				(n = 17)				(n = 13)					
Educational level														
High school+	0.827 (0.104)	0.8–0.855	0.002	0.535	0.863 (0.061)	0.835–0.891	0.002	1.138	0.808 (0.118)	0.768–0.848	0.074	0.417	0.057	0.586
	(n = 56)				(n = 20)				(n = 36)					
High school−	0.754 (0.13)	0.716–0.791			0.746 (0.132)	0.652–0.841			0.756 (0.131)	0.713–0.798			0.842	0.076
	(n = 49)				(n = 10)				(n = 39)					
Employment status														
Employed	0.807 (0.114)	0.782–0.833	0.049	0.422	0.848 (0.071)	0.817–0.878	0.01	0.941	0.79 (0.125)	0.756–0.823	0.372	0.232	0.037	0.571
	(n = 79)				(n = 24)				(n = 55)					
Unemployed/had unstable employment	0.754 (0.136)	0.7–0.808			0.737 (0.151)	0.598–0.877			0.76 (0.134)	0.697–0.823			0.718	0.161
	(n = 27)				(n = 7)				(n = 20)					
CHD type														
Simple	0.8 (0.119)	0.776–0.825	0.08	0.459	0.829 (0.097)	0.791–0.866	0.053	0.872	0.788 (0.126)	0.757–0.819	0.349	0.299	0.131	0.365
	(n = 93)				(n = 28)				(n = 65)					
Complex PAH	0.743 (0.129)	0.674–0.812			0.729 (0.13)	0.568–0.89			0.749 (0.135)	0.659–0.84			0.786	0.151
	(n = 16)				(n = 5)				(n = 11)					
CHD treatment														
Repaired	0.796 (0.117)	0.771–0.821	0.501	0.149	0.819 (0.093)	0.783–0.855	0.5	0.257	0.785 (0.126)	0.752–0.818	0.772	0.077	0.206	0.307
	(n = 86)				(n = 28)				(n = 58)					
Unrepaired/palliative	0.777 (0.138)	0.717–0.836			0.783 (0.175)	0.567–1.0			0.775 (0.133)	0.709–0.841			0.905	0.051
	(n = 23)				(n = 5)				(n = 18)					
EuroQOL Visual Analogue Scale (EQ-VAS)														
Total	66.3 (12.5)	63.9–68.7	-	-	66.9 (12.6)	62.4–71.4	-	-	66.1 (12.6)	63.2–68.9	-	-	0.754	0.016
	(n = 109)				(n = 33)				(n = 76)					
Age groups														
16–30	69.9 (10.8)	66.4–73.4	0.024	0.466	72.1 (10.2)	67.0–77.1	0.008	0.985	68.1 (11.2)	63.0–73.2	0.385	0.23	0.228	0.373
	(n = 39)				(n = 18)				(n = 21)					
31+	64.3 (13.1)	61.2–67.4			60.7 (12.8)	53.6–67.8			65.3 (13.1)	61.7–68.8			0.259	0.355
	(n = 70)				(n = 15)				(n = 55)					
Marital status														
Married	67.0 (11.2)	64.4–69.5	0.376	0.177	64.4 (12.1)	57.9–70.8	0.276	0.382	67.6 (11.0)	64.8–70.4	0.016	0.638	0.306	0.277
	(n = 79)				(n = 16)				(n = 63)					
Unmarried	64.6 (15.5)	58.8–70.4			69.2 (13.0)	62.5–75.9			58.5 (16.9)	48.3–68.7			0.058	0.71
	(n = 30)				(n = 17)				(n = 13)					
Educational level														
High school+	69.5 (12.0)	66.3–72.7	0.008	0.527	71.9 (9.1)	67.6–76.1	0.015	0.937	68.2 (13.3)	63.7–72.7	0.124	0.361	0.279	0.325
	(n = 56)				(n = 20)				(n = 36)					
High school−	63.2 (11.9)	59.7–66.6			61.0 (13.7)	51.2–70.8			63.7 (11.6)	60.0–67.5			0.526	0.213
	(n = 49)				(n = 10)				(n = 39)					
Employment status														
Employed	68.4 (12.5)	65.6–71.2	0.009	0.627	71.1 (9.6)	67.1–75.2	0.009	1.067	67.2 (13.5)	63.5–70.8	0.134	0.431	0.2	0.333
	(n = 79)				(n = 24)				(n = 55)					
Unemployed/had unstable employment	61.3 (10.0)	57.4–65.2			58.6 (13.5)	46.1–71.0			62.3 (8.7)	58.2–66.3			0.411	0.326
	(n = 27)				(n = 7)				(n = 20)					
CHD type														
Simple	67.4 (12.0)	64.9–69.9	0.029	0.565	68.8 (12.0)	64.2–73.5	0.034	1.094	66.8 (12.0)	63.8–69.8	0.23	0.362	0.452	0.167
	(n = 93)				(n = 28)				(n = 65)					
Complex/PAH	60.0 (14.1)	52.5–67.5			56.0 (11.4)	41.8–70.2			61.8 (15.4)	51.5–72.2			0.465	0.428
	(n = 16)				(n = 5)				(n = 11)					
CHD treatment														
Repaired	66.9 (12.6)	64.2–69.6	0.352	0.225	67.4 (11.8)	62.8–72.0	0.588	0.222	66.6 (13.1)	63.2–70.1	0.47	0.199	0.797	0.064
	(n = 86)				(n = 28)				(n = 58)					
Unrepaired/palliative	64.1 (12.3)	58.8–69.5			64.0 (18.2)	41.4–86.6			64.2 (10.9)	58.8–69.6			0.979	0.013
	(n = 23)				(n = 5)				(n = 18)					

CHD, Congenital heart disease; PAH, Pulmonary artery hypertension; SD, Standard Deviation; CI, Confidence Interval; Employed status included stable employment, homemaker, retired, student or other works; * Compared between men and women; Cohen's d is the appropriate effect size measure; Using independent samples t-test

Table 3 also shows that EQ-VAS in patients who were aged > 30 years was lower than in those aged ≤ 30 years (64.3 [SD = 13.1], 95% CI: 61.2–67.4 vs 69.9 [SD = 10.8], 95% CI: 66.4–73.4, *p* = 0.024 overall; 60.7 [SD = 12.1], 95% CI: 53.6–67.8 vs 72.2 [SD = 10.2], 95% CI: 67.0–77.1, *p* = 0.008 in men; using the Student *t*-test; respectively), EQ-VAS in patients with an educational level lower than high school was lower than in those with an educational level of high school or higher (63.2 [SD = 11.9], 95% CI: 59.7–66.6 vs 69.5 [SD = 12.0], 95% CI: 66.3–72.7, *p* = 0.008 overall; 61.0 [SD = 13.7], 95% CI: 51.2–70.8 vs 71.9 [SD = 9.1], 95% CI: 67.6–76.1, *p* = 0.015 in men; using the Student t-test; respectively), EQ-VAS in patients with an unemployed status/unstable employment was lower than in those with an employed status (61.3 [SD = 10.0], 95% CI: 57.4–65.2 vs 68.4 [SD = 12.5], 95% CI, 65.6–71.2, *p* = 0.009 overall; 58.6 [SD = 13.5], 95% CI: 46.1–71.1 vs 71.1 [SD = 9.6], 95% CI: 67.1–75.2, *p* = 0.009 in men; using Student t-test; respectively), EQ-VAS in patients with complex CHD/PAH was lower than in those with simple CHD (60.0 [SD = 14.1], 95% CI: 52.5–67.5 vs 67.4 [SD = 12.0], 95% CI: 64.9–69.9, *p* = 0.029 overall; 56.0 [SD = 11.4], 95% CI: 41.8–70.2 vs 68.8 [SD = 12.0], 95% CI: 64.2–73.5, *p* = 0.034 in men; using the Student t-test; respectively). Moreover, EQ-DS in unmarried women was 58.5 (SD = 16.9, 95% CI: 48.3–68.7), which was lower than that in married women (67.6; SD = 11.0, 95% CI: 64.8–70.4), with *p* = 0.016, using the Student t-test.

Table 4 compares SWLS, HADS-A, and HADS-D among subgroups. Specifically, the mean of SWLS of patients with an unemployed status/unstable employment was lower than that of patients with an employed status. Based on the HADS classification, mean of HADS-A and HADS-D in patients aged > 30 years, had educational level at high school and above, had an unemployed status/unstable employment, and had complex CHD/PAH were lower than those in patients aged ≤ 30 years, had an educational level of high school and above, had an employed status, and had simple CHD, respectively. These differences were statistically significant (*p* = 0.001, *p* = 0.001, *p* = 0.007, *p* = 0.032 for HADS-A; *p* = 0.002, *p* = 0.001, *p* = 0.004, *p* = 0.028 for HADS-D; respectively).

**Table 4:** SWLS, HADS-A and HADS-D by subgroups in adults with congenital heart disease

Groups	Total						Men					
	Median	IQR	U	Z	*p* value	Cohen’s d	Median	IQR	U	Z	*p* value	Cohen’s d
Satisfaction with Life Scale (SWLS)												
Total	25.0	5.0	–	–	–	–	26.0	4.5	–	–	–	–
	(n = 109)						(n = 33)					
Age groups												
16–30	26.0	4.0	1226.5	− 0.88	0.379	0.168	26.5	5.25	124.5	− 0.384	0.708	0.132
	(n = 39)						(n = 18)					
31+	25.0	5.0					26.0	4.0				
	(n = 70)						(n = 15)					
Marital status												
Married	25.0	5.0	911.5	− 1.865	0.062	0.361	26.5	4.0	96.0	− 1.457	0.157	0.518
	(n = 79)						(n = 16)					
Unmarried	25.0	6.25					26.0	6.5				
	(n = 30)						(n = 17)					
Educational level												
High school+	26.0	4.75	1108.5	− 1.7	0.089	0.335	26.5	4.0	79.0	− 0.935	0.373	0.342
	(n = 56)						(n = 20)					
High school−	25.0	5.0					25.5	8.25				
	(n = 49)						(n = 10)					
Employment status												
Employed	26.0	5.0	779.5	− 2.09	0.037	0.413	27.0	4.0	53.5	− 1.457	0.153	0.536
	(n = 79)						(n = 24)					
Unemployed/had unstable employment	24.0	7.0					25.0	11.0				
	(n = 27)						(n = 7)					
CHD type												
Simple	25.0	5.0	632.0	− 0.964	0.335	0.184	26.5	4.75	58.0	− 0.609	0.575	0.211
	(n = 93)						(n = 28)					
Complex/PAH	25.0	4.75					25.0	4.5				
	(n = 16)						(n = 5)					
CHD treatment												
Repaired	26.0	5.0	933.0	− 0.418	0.676	0.08	26.0	2.0	59.5	− 0.533	0.609	0.184
	(n = 23)						(n = 5)					
Unrepaired/palliative	25.0	5.0					26.5	5.5				
	(n = 86)						(n = 28)					
Hospital Anxiety and Depression Scale-Anxiety subscale (HADS-A)												
Total	6.0	7.0	–	–	–	–	4.0	5.0	–	–	–	–
	(n = 107)						(n = 31)					
Age groups												
16–30	3.5	6.25	824.5	− 3.179	0.001	0.643	3.0	4.5	68.0	− 2.041	0.044	0.781
	(n = 38)						(n = 17)					
31+	7.0	5.0					5.5	6.25				
	(n = 69)						(n = 14)					
Marital status												
Married	6.5	6.0	1064.5	− 0.468	0.64	0.09	5.0	5.0	113.0	− 0.279	0.8	0.1
	(n = 78)						(n = 15)					
Unmarried	4.0	10.0					3.5	5.75				
	(n = 29)						(n = 16)					
Educational level												
High school+	5.0	5.0	798.5	− 3.44	0.001	0.717	3.0	3.75	23.5	− 2.903	0.003	1.293
	(n = 56)						(n = 20)					
High school−	9.0	7.0					9.0	9.25				
	(n = 47)						(n = 8)					
Employment status												
Employed	6.0	6.0	655.0	− 2.706	0.007	0.548	3.0	5.0	29.0	− 2.174	0.031	0.873
	(n = 78)						(n = 23)					
Unemployed/had unstable employment	9.5	7.5					9.5	11.25				
	(n = 26)						(n = 6)					
CHD type												
Simple	6.0	6.75	451.5	− 2.148	0.032	0.423	4.0	5.0	38.5	− 0.921	0.376	0.333
	(n = 92)						(n = 27)					
Complex/PAH	10.0	7.0					9.0	13.75				
	(n = 15)						(n = 4)					
CHD treatment												
Repaired	6.0	8.25	876.5	− 0.453	0.651	0.087	4.0	10.25	52.5	− 0.089	0.932	0.032
	(n = 22)						(n = 4)					
Unrepaired/palliative	7.0	7.0					4.0	5.0				
	(n = 85)						(n = 27)					
Hospital Anxiety and Depression Scale-Depression subscale (HADS-D)												
Total	5.0	5.0	–	–	–	–	4.0	5.0	–	–	–	–
	(n = 109)						(n = 33)					
Age groups												
16–30	4.0	4.0	878.0	− 3.096	0.002	0.617	2.5	3.5	69.0	− 2.407	0.016	0.913
	(n = 39)						(n = 18)					
31+	6.0	5.25					6.0	4.0				
	(n = 70)						(n = 15)					
Marital status												
Married	5.0	5.0	1144.0	− 0.28	0.78	0.053	5.0	4.75	119.0	− 0.618	0.557	0.214
	(n = 79)						(n = 16)					
Unmarried	4.0	8.0					3.0	5.5				
	(n = 30)						(n = 17)					
Educational level												
High school+	4.0	3.0	846.5	− 3.395	0.001	0.698	3.0	2.0	41.0	− 2.622	0.008	1.076
	(n = 56)						(n = 20)					
High school−	7.0	6.0					7.5	7.25				
	(n = 49)						(n = 10)					
Employment status												
Employed	4.0	4.0	673.0	− 2.87	0.004	0.577	3.0	3.75	43.0	− 1.955	0.054	0.742
	(n = 79)						(n = 24)					
Unemployed/had unstable employment	8.0	8.0					8.0	11.0				
	(n = 27)						(n = 7)					
CHD type												
Simple	4.0	5.0	489.5	− 2.191	0.028	0.427	3.0	5.0	36.5	− 1.696	0.093	0.612
	(n = 93)						(n = 28)					
Complex/PAH	7.0	6.0					6.0	9.5				
	(n = 16)						(n = 5)					
CHD treatment												
Repaired	6.0	7.0	796.5	− 1.438	0.151	0.276	3.0	7.0	56.5	0.494	0.509	0.238
	(n = 23)						(n = 5)					
Unrepaired/palliative	5.0	5.0					4.0	5.0				
	(n = 86)						(n = 28)					

Groups	Women						U*	Z*	*p* value*	Cohen’s d*
	Median	IQR	U	Z	*p* value	Cohen’s d				
Satisfaction with Life Scale (SWLS)										
Total	25.0	4.75	–	–	–	–	1075.5	–1.183	0.237	0.227
	(n = 76)									
Age groups										
16–30	26.0	3.5	481.5	− 1.12	0.263	0.258	183	− 0.17	0.878	0.054
	(n = 21)									
31+	25.0	5.0					314.0	− 1.417	0.157	0.342
	(n = 55)									
Marital status										
Married	25.0	5.0	276.5	− 1.843	0.065	0.431	368.5	− 1.663	0.096	0.379
	(n = 63)									
Unmarried	24.0	6.5					83.0	− 1.157	0.263	0.43
	(n = 13)									
Educational level										
High school+	26.0	5.0	595.0	− 1.14	0.254	0.264	312.5	− 0.817	0.414	0.218
	(n = 36)									
High school−	24.0	4.0					181.0	− 0.349	0.742	0.099
	(n = 39)									
Employment status										
Employed	25.0	5.0	438.0	− 1.348	0.178	0.314	528.5	− 1.41	0.159	0.319
	(n = 55)									
Unemployed/had unstable employment	24.0	6.25					69.5	− 0.028	0.978	0.011
	(n = 20)									
CHD type										
Simple	25.0	5.0	302.5	− 0.816	0.415	0.187	792.0	− 0.993	0.321	0.206
	(n = 65)									
Complex/PAH	24.0	5.0					24.0	0.689	0.743	0.199
	(n = 11)									
CHD treatment										
Repaired	25.5	6.0	466.0	− 0.687	0.492	0.157	43.0	− 0.15	0.914	0.062
	(n = 18)									
Unrepaired/palliative	25.0	4.0					669.0	− 1.325	0.185	0.287
	(n = 58)									
Hospital Anxiety and Depression Scale-Anxiety subscale (HADS-A)										
Total	7.0	6.0		–	–	–	745.5	− 2.982	0.003	0.6
	(n = 76)									
Age groups										
16–30	6.0	7.5	440.0	− 1.603	0.109	0.373	113.0	− 1.944	0.056	0.657
	(n = 21)									
31+	8.0	5.0					298.0	− 1.303	0.193	0.316
	(n = 55)									
Marital status										
Married	7.0	6.0	317.5	− 1.274	0.203	0.294	306.0	− 2.119	0.034	0.492
	(n = 63)									
Unmarried	12.0	12.5					62.5	− 1.835	0.068	0.718
	(n = 13)									
Educational level										
High school+	7.0	7.0	551.0	− 1.607	0.108	0.376	163.5	− 3.38	0.001	1.005
	(n = 36)									
High school−	8.0	7.0					155.5	− 0.014	0.989	0.004
	(n = 39)									
Employment status										
Employed	7.0	7.0	421.5	− 1.545	0.122	0.361	340.0	− 3.221	0.001	0.779
	(n = 55)									
Unemployed/had unstable employment	9.5	6.75					58.5	− 0.092	0.929	0.036
	(n = 20)									
CHD type										
Simple	7.0	6.5	242.0	− 1.711	0.087	0.399	540.5	− 2.905	0.004	0.632
	(n = 65)									
Complex/PAH	10.0	6.0					21.0	− 0.131	0.949	0.067
	(n = 11)									
CHD treatment										
Repaired	6.0	7.5	520.0	− 0.025	0.98	0.006	22.5	− 1.159	0.262	0.505
	(n = 18)									
Unrepaired/palliative	7.5	6.0					490.0	− 2.777	0.005	0.629
	(n = 58)									
Hospital Anxiety and Depression Scale-Depression subscale (HADS-D)										
Total	5.0	5.0		–	–	–	914.0	− 2.255	0.024	0.555
	(n = 76)									
Age groups										
16–30	4.0	4.0	438.0	− 1.631	0.103	0.378	116.5	− 2.064	0.04	0.692
	(n = 21)									
31+	6.0	6.0					377.5	− 0.503	0.615	
	(n = 55)									
Marital status										
Married	5.0	4.0	307.5	− 1.416	0.157	0.327	420.0	− 1.032	0.302	0.232
	(n = 63)									
Unmarried	10.0	10.5					60.5	− 2.104	0.035	0.827
	(n = 13)									
Educational level										
High school+	4.0	3.75	519.5	− 1.948	0.051	0.459	188.0	− 2.975	0.003	0.855
	(n = 36)									
High school−	7.0	6.0					191.5	− 0.087	0.932	0.025
	(n = 39)									
Employment status										
Employed	4.0	5.0	378.0	− 2.074	0.038	0.49	411.5	− 2.674	0.007	0.624
	(n = 55)									
Unemployed/ had unstable employment	8.0	7.5					65.5	− 0.251	0.808	0.096
	(n = 20)									
CHD type										
Simple	5.0	5.5	260.5	− 1.441	0.15	0.333	630.5	− 2.355	0.019	0.5
	(n = 65)									
Complex/PAH	8.0	6.0					26.0	− 0.172	0.913	0.085
	(n = 11)									
CHD treatment										
Repaired	6.5	6.0	433.0	− 1.094	0.274	0.251	31.5	− 1.015	0.325	0.429
	(n = 18)									
Unrepaired/palliative	5.0	5.0					610.5	− 1.868	0.062	0.409
	(n = 58)									

CHD: Congenital heart disease; PAH: Pulmonary artery hypertension; IQR: Interquartile Range; Employed status included stable employment, homemaker, retired, student or other works; Using independent samples Mann–Whitney-U test; Cohen's d is the appropriate effect size measure; *Compared between men and women

**Fig. 1:**
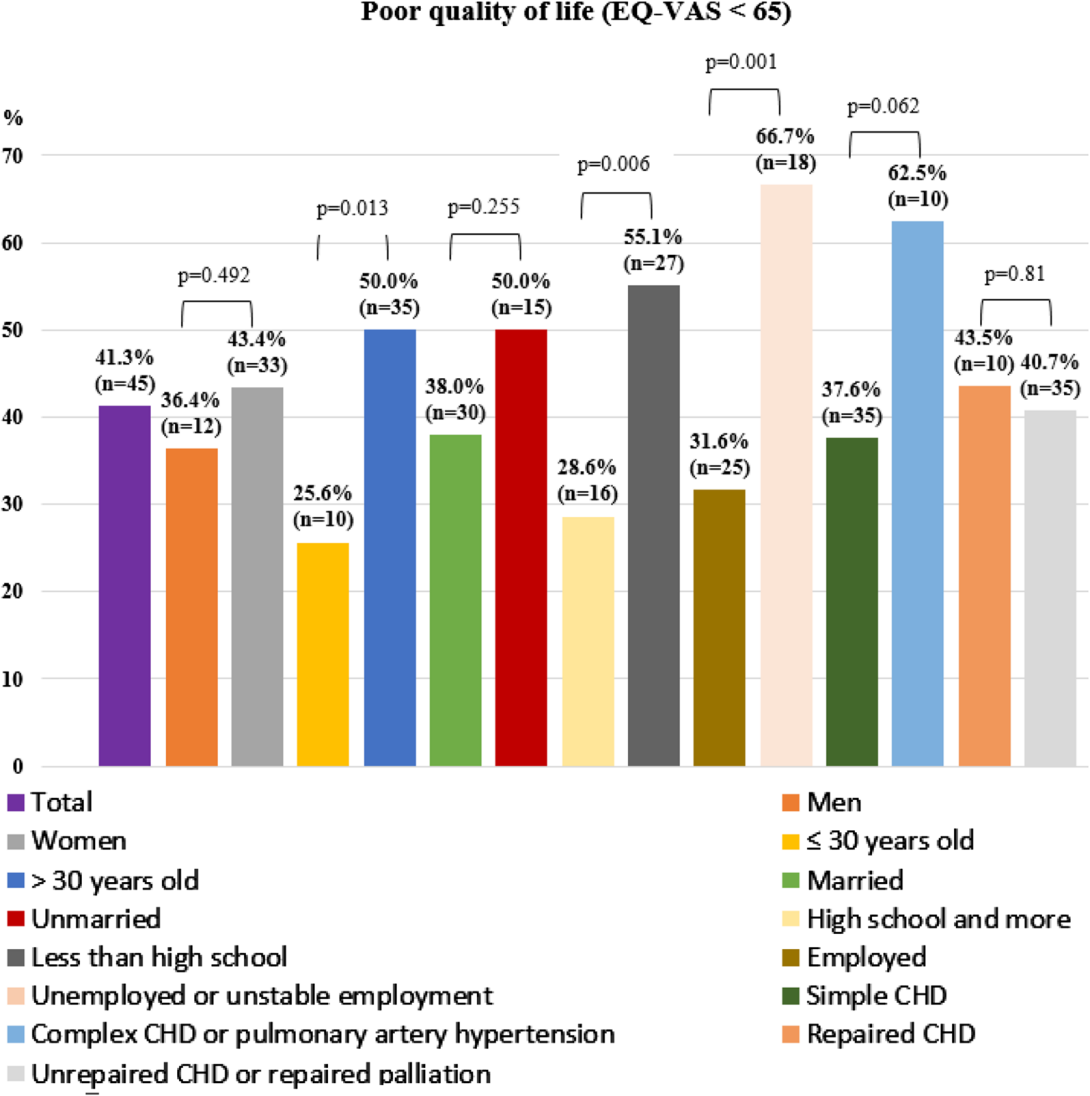
Poor quality of life by subgroups in adults with congenital heart disease. Poor quality of life (QOL) was defined as EQ-VAS < 65. The prevalence of poor QOL in patients who were aged > 30 years was higher than those aged ≤ 30 years (50%, n = 35 vs 25.6%, n = 10, *p* = 0.013; using the Chi-square test; respectively). The prevalence of poor QOL in patients with an educational level of high school and below was higher than in those with an educational level of high school and above (55.1%, n = 27 vs 28.6%, n = 16, *p* = 0.006; using Chi-square test; respectively). The prevalence of poor QOL in patients with an unemployed status/unstable employment was higher than in those with an employed status (66.7%, n = 18 vs 37.6%, n = 35, *p* = 0.001; using Chi-square test; respectively). CHD: Congenital heart disease; EQ-VAS: EuroQOL Visual Analogue Scale

Figure 1 shows the prevalence of poor QOL, defined as EQ-VAS < 65, by subgroup. The prevalence of poor QOL in patients who were aged > 30 years was higher than those aged ≤ 30 years (50%, n = 35 vs 25.6%, n = 10, *p* = 0.013; using the Chi-square test; respectively). The prevalence of poor QOL in patients with an educational level of high school and below was higher than in those with an educational level of high school and above (55.1%, n = 27 vs 28.6%, n = 16, *p* = 0.006; using Chi-square test; respectively). The prevalence of poor QOL in patients with an unemployed status/unstable employment was higher than in those with an employed status (66.7%, n = 18 vs 37.6%, n = 35, *p* = 0.001; using Chi-square test; respectively).

**Fig. 2:**
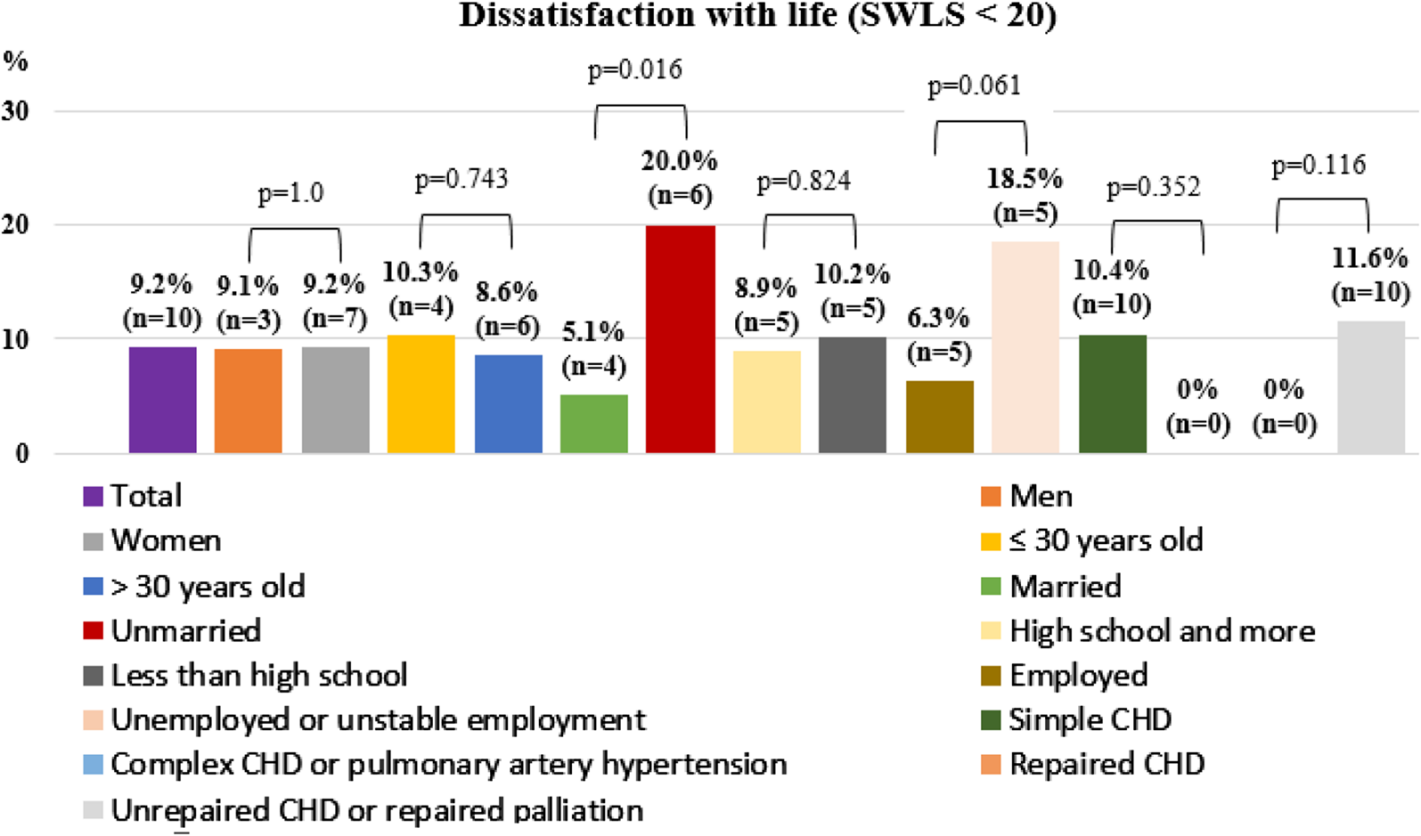
Life dissatisfaction by subgroups in adults with congenital heart disease. Life dissatisfaction was defined as SWLS < 20. The prevalence of life dissatisfaction in unmarried patients was higher than in married patients (20%, n = 6 vs 5.1%, n = 4, *p* = 0.016; using the Fisher’s exact test; respectively). CHD: Congenital heart disease; SWLS: Satisfaction with Life Scale

Figure 2 shows the prevalence of life dissatisfaction by subgroup. Notably, the prevalence of life dissatisfaction in unmarried patients was higher than in married patients (20%, n = 6 vs 5.1%, n = 4, *p* = 0.016; using the Fisher’s exact test; respectively).

**Fig. 3:**
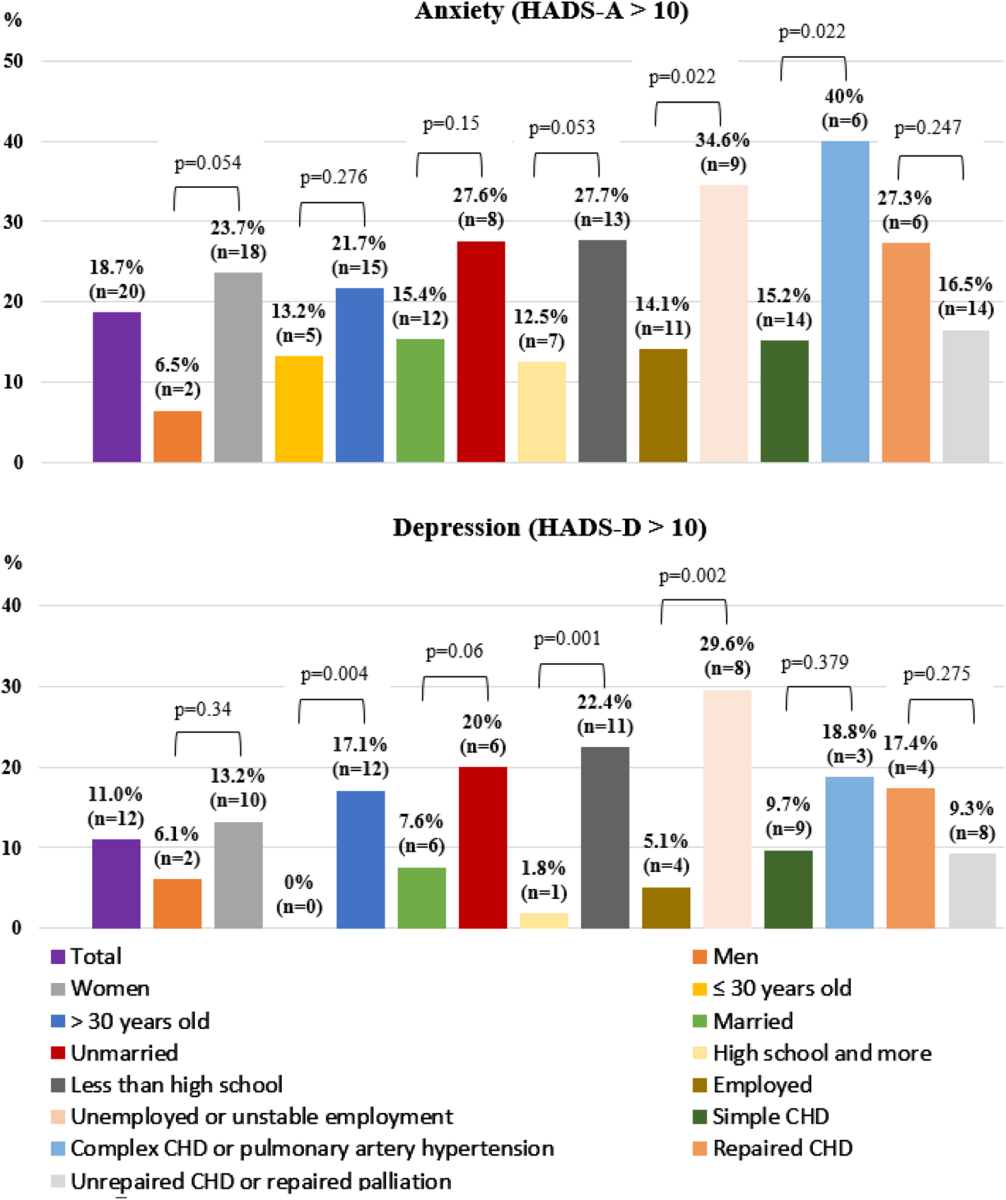
Symptoms of anxiety and depression by subgroups in adults with congenital heart disease. Anxiety was defined as HADS-A > 10. Depression was defined as HADS-D > 10. The prevalence of anxiety in patients with an unemployed status/unstable employment was higher than in those with an employed status (34.6%, n = 9 vs 14.1%, n = 11, *p* = 0.022; using Chi-square test; respectively). The prevalence of depression in patients with an unemployed status/unstable employment was higher than in those with an employed status (29.6%, n = 8 vs 5.1%, n = 4, *p* = 0.002; using the Fisher’s exact test; respectively). The prevalence of anxiety in patients who had complex CHD/PAH is higher than in those with simple CHD (40%, n = 6 vs 15.2%, n = 14, *p* = 0.022; using Chi-square test; respectively). The prevalence of depression of patients aged > 30 years is higher than that of patients aged ≤ 30 years (17.1%, n = 12 vs 0%, n = 0, *p* = 0.004; using Fisher’s exact test; respectively). The prevalence of depression in patients with an educational level of high school and below was higher than in those with an educational level of high school and above (22.4%, n = 11 vs 1.8%, n = 1, *p* = 0.001; using Fisher’s exact test; respectively). CHD: Congenital heart disease; HADS-A: Hospital Anxiety and Depression Scale-Anxiety subscale; HADS-D: Hospital Anxiety and Depression Scale-Depression subscale; PAH: Pulmonary artery hypertension

Figure 3 shows the prevalence of anxiety and depression by subgroup. The prevalence of anxiety in patients with an unemployed status/unstable employment was higher than in those with an employed status (34.6%, n = 9 vs 14.1%, n = 11, *p* = 0.022; using Chi-square test; respectively). The prevalence of depression in patients with an unemployed status/unstable employment was higher than in those with an employed status (29.6%, n = 8 vs 5.1%, n = 4, *p* = 0.002; using the Fisher’s exact test; respectively). The prevalence of anxiety in patients who had complex CHD/PAH is higher than in those with simple CHD (40%, n = 6 vs 15.2%, n = 14, *p* = 0.022; using Chi-square test; respectively). The prevalence of depression of patients aged > 30 years is higher than that of patients aged ≤ 30 years (17.1%, n = 12 vs 0%, n = 0, *p* = 0.004; using Fisher’s exact test; respectively). The prevalence of depression in patients with an educational level of high school and below was higher than in those with an educational level of high school and above (22.4%, n = 11 vs 1.8%, n = 1, *p* = 0.001; using Fisher’s exact test; respectively).

The results of the stratified logistic regressions predicting poor QOL and health status are summarized in Table 5. Multivariable logistic regression showed that poor QOL, defined as EQ-VAS < 65, was associated with being unemployed/unstable employment (OR = 4.43, 95% CI 1.71–11.47, *p* = 0.002). Life dissatisfaction was associated with being unmarried (OR = 4.63, 95% CI 1.2–17.86, *p* = 0.026). Anxiety was associated with being unemployed/unstable employment (OR = 3.88, 95% CI 1.27–11.84, *p* = 0.017) and having a complex CHD/PAH (OR = 4.84, 95% CI 1.33–17.54, *p* = 0.016). Moreover, depression was associated with being unemployed/unstable employment (OR = 4.63, 95% CI 1.22–17.59, *p* = 0.025).

**Table 5:** Univariable and multivariable predictors of poor quality of life and health status in adults with congenital heart disease on logistic regression

Independent variables	Beta	Standard error	*p* value (Wald statistic)	OR (95%CI)
Univariable logistic regression				
Poor quality of life				
Women	0.295	0.43	0.492	1.34 (0.58; 3.12)
> 30 years old	1.065	0.438	0.015	2.9 (1.23; 6.84)
Unmarried	0.491	0.433	0.257	1.63 (0.7; 3.81)
Less than high school	1.121	0.412	0.007	3.07 (1.37; 6.88)
Unemployed/ had unstable employment	1.463	0.475	0.002	4.3 (1.7; 10.95)
Complex CHD or PAH	1.016	0.559	0.069	2.76 (0.92; 8.26)
Unrepaired CHD or repaired palliation	0.114	0.474	0.81	1.12 (0.44; 2.84)
Life dissatisfaction				
Women	0.014	0.724	0.984	1.01 (0.25; 4.19)
> 30 years old	− 0.198	0.679	0.77	0.82 (0.22; 3.1)
Unmarried	1.545	0.687	0.024	4.69 (1.22; 18.01)
Less than high school	0.148	0.665	0.824	1.16 (0.32; 4.27)
Unemployed/had unstable employment	1.213	0.677	0.073	3.36 (0.89; 12.69)
Complex CHD or PAH	19.087	10048.242	0.998	–
Unrepaired CHD or repaired palliation	19.175	8380.813	0.998	–
Anxiety				
Women	1.504	0.779	0.054	4.5 (0.98; 20.73)
> 30 years old	0.606	0.562	0.281	1.83 (0.61; 5.51)
Unmarried	0.74	0.521	0.155	2.1 (0.76; 5.81)
Less than high school	0.984	0.519	0.058	2.68 (0.97; 7.41)
Unemployed/had unstable employment	1.171	0.525	0.026	3.23 (1.15; 9.03)
Complex CHD or PAH	1.312	0.602	0.029	3.71 (1.14; 12.08)
Unrepaired CHD or repaired palliation	0.643	0.561	0.252	1.9 (0.63; 5.71)
Depression				
Women	0.854	0.805	0.289	2.35 (0.49; 11.37)
> 30 years old	19.627	6436.026	0.998	–
Unmarried	1.112	0.623	0.074	3.04 (0.9; 10.32)
Less than high school	2.768	1.066	0.009	15.92 (1.97; 128.52)
Unemployed/ had unstable employment	2.066	0.664	0.002	7.9 (2.15; 29.01)
Complex CHD or PAH	0.767	0.73	0.293	2.15 (0.52; 9.01)
Unrepaired CHD or repaired palliation	0.719	0.664	0.279	2.05 (0.56; 7.54)
Multivariable logistic regression				
Poor quality of life				
*Step 1*				
Unemployed/ had unstable employment	1.426	0.475	0.003	4.16 (1.64; 10.56)
Constant	-0.693	0.408	0.09	-
*Step 2*				
Unemployed/ had unstable employment	1.487	0.486	0.002	4.43 (1.71; 11.47)
Complex CHD or PAH	1.138	0.587	0.053	3.12 (0.99; 9.87)
Constant	− 1.695	0.676	0.012	–
Life dissatisfaction				
*Step 1*				
Unmarried	1.533	0.689	0.026	4.63 (1.2; 17.86)
Constant	1.344	0.458	0.003	–
Anxiety defined				
*Step 1*				
Unemployed/ had unstable employment	1.141	0.526	0.03	3.13 (1.12; 8.76)
Constant	0.636	0.412	0.123	–
*Step 2*				
Unemployed/ had unstable employment	1.29	0.552	0.02	3.63 (1.23; 10.72)
Complex CHD or PAH	1.43	0.636	0.024	4.19 (1.2; 14.56)
Constant	− 0.616	0.701	0.379	–
*Step 3*				
Women	1.62	0.849	0.056	5.05 (0.96; 26.67)
Unemployed/ had unstable employment	1.36	0.57	0.017	3.88 (1.27; 11.84)
Complex CHD or PAH	1.58	0.657	0.016	4.84 (1.33; 17.54)
Constant				–
Depression defined as HADS-D > 10				
*Step 1*				
Unemployed/ had unstable employment	2.039	0.664	0.002	7.68 (2.09; 28.25)
Constant	0.865	0.421	0.04	–
*Step 2*				
> 30 years old	19.192	6332.88	0.998	–
Unemployed/ had unstable employment	1.531	0.682	0.025	4.63 (1.22; 17.59)
Constant	0.693	0.433	0.109	–

CHD: Congenital heart disease; PAH: Pulmonary artery hypertension; OR: Odds Ratio; CI: Confidence Interval. Poor quality of life was defined as EQ-VAS < 65; Life dissatisfaction was defined as SWLS < 20; Anxiety was defined as HADS-A > 10; Depression was defined as HADS-D > 10

## Discussion

To the best of our knowledge, this study is the first to provide evidence regarding QOL and health status in adults with CHD in Vietnam, thus contributing to the essential knowledge on these matters in Vietnam. Hospitalized adults with CHD had reduced scores of QOL as measured using EQ-5D-5L. A significant number of hospitalized adults with CHD reported poor QOL, anxiety, and depression. Notably, we found that poor QOL and psychological problems were significantly related to biological and social characteristics including gender, age, marital status, education level, employment status, and CHD type in these patients.

### Characteristic participants

In our study, 69.7% of adults with CHD were women. A recent study in Quebec indicated a higher prevalence of women among the patients with CHD (57%) [27]. Female predominance was observed in simple CHD such as atrial septal defect, ventricular septal defect, and patent ductus arteriosus. In particular, women constituted about 60–65% of patients with atrial septal defect [27, 28]. Similarly, 85.3% of the patients in our study had simple CHD and 48.6% an isolated atrial septal defect. A recent study in Vietnam reported that the prevalence of atrial septal defect among patients with CHD increased from children to adults (12.3% aged 0–9 years, 28.7% aged 10–19 years, 49.6% aged 20–39 years and 70.8% aged 40 years) [29].

We also noted that the rate of young patients, married/cohabiting patients, and patients with an education level of high school and above in our study was similar to that of the general population reported by the General Statistics Office of Vietnam in 2019 (http://tongdieutradanso.vn/trang-chu.html): 35.8% vs 29.7%, 72.5% vs 69.2%, and 55% vs 36.6%, respectively.

Our patients presented a higher rate of unemployment than the general Vietnamese population (5.5% vs 2.04% in 2019, respectively; https://data.worldbank.org/). The high rate of unemployment in adults with CHD was also described in a recent study in Germany, amounting to 7.8% [30]. Furthermore, employment rate in our study also revealed a high rate of unstable employment (19.3%), suggesting a relationship between employment status and QOL and health status in adults with CHD.

### Quality of life characteristics

Our findings demonstrated a high rate of poor QOL, as measured using EQ-5D-5L, in hospitalized adults with CHD in Vietnam. The participants of our study showed lower mean EQ-DS and EQ-VAS scores compared to healthy individuals from a general population study in Vietnam [31] (for EQ-DS, 0.792 [SD = 0.121] vs 0.91 [SD = 0.15], and for EQ-VAS, 66.3 [SD = 12.5] vs 87.4 [SD = 14.3], respectively). We noted that, in this study, most of the Vietnamese general population comprised women (61.5%), youths (aged < 35, 54.2%), and people living with their spouse/partner (65.5%) [31]. Similarly, of our patients, 69.7% were women, 35.8% were aged < 31, and 72.5% were married/cohabiting. Compared to our results, other studies had also reported a tendency of reduced EQ-DS and EQ-VAS scores among Vietnamese patients with chronic cardiovascular diseases [32, 33]. CHD is a chronic condition; if it is under-diagnosed or under-treated, it can result in reduced QOL and may even be life-threatening [4].

We highlighted reduced scores of QOL and health status in patients from Vietnam—a typical developing country—similar to the findings from previous studies in Iran [15]. Indeed, our participants with CHD had greater problems with pain/discomfort, anxiety/depression, mobility, and usual activities, similar to the results of a previous study [34]. Meanwhile, a previous systematic review and meta-analysis of 18 studies from developed countries found that QOL in young adults with CHD was comparable or better compared with the healthy controls [4]. This result may be explained by the differences in economic factors, educational status, cultural factors, and medical quality between developing and developed countries [10]. A report from APPROACH-IS on 15 countries observed that patient with CHD in countries with a lower power distance index and higher individualism tended to have a stronger sense of coherence. Further, a stronger sense of coherence positively contributed to QOL in adults with CHD [35]. Besides, high standards of living, which was expressed as high Gross Domestic Product per capita, high Human Development Index, and a good healthcare system, were common in developed countries but limited in developing countries. The APPROACH-IS report also noted that adults with CHD in countries with a higher standard of living had better physical and psychological health than those in countries with lower standards of living [36].

### Life satisfaction

The prevalence of life dissatisfaction was low in adults with CHD in Vietnam (9.2%), with a high mean score of satisfaction of 25.2 points. This life satisfaction score appeared to be better than that recently reported in another developing country, Iran [15], but was similar to that of the 15 countries reported in APPROACH-IS [10]. Consistent with Vietnamese adults with other complex diseases such as spinal cord injury [37], our patients with CHD also presented trends of positive cognitive judgments about their life. Notably, positive thinking about life was common among the Vietnamese population, which ranked fifth out of 140 countries worldwide and second in the Asia Pacific region in the Happy Planet Index of 2019 (http://happyplanetindex.org/). Life satisfaction of one individual was influenced by specific country variables such as economic status, cultural factors, and social characteristics. Level of life satisfaction was more strongly associated with financial satisfaction in low-and middle-income countries than in high-income countries [38]. Likewise, life satisfaction of individuals in Asian countries was strongly affected by a society's national integration [39]. It explained the trending life satisfaction in patients living in a country in Asia with a dynamic economy and culture such as Vietnam.

### Anxiety and depression of adults with congenital heart disease

We noted the high prevalence of anxiety and depression in adults with CHD. In Vietnam, the prevalence of self-reported anxiety among adults with CHD is more than seven times higher than that of the general population (18.7% vs. 2.6%, respectively), while the prevalence of self-reported depression among adults with CHD is more than four times higher than that of the general population (11% vs 2.8%, respectively) [40]. The prevalence of anxiety and depression in our patients is similar to that in Vietnamese adults with cancer (18.7% vs 15.5%, respectively) [26]. Our findings were similar to that of a recent study, which reported that 30.7% of adults with CHD had mood disorders and 28% had anxiety disorders [20]. However, the exact prevalence of psychological disturbances, their geographical differences, and valid scales of evaluations are lacking. Based on the literature, adults with CHD constitute a high-risk group for developing emotional problems related to reduced exercise capacity, complications, and social barriers [5, 20, 41–44]. Therefore, screening for mental disorders in adults with CHD is important for early diagnosis and appropriate treatment because they require special medical attention and psychosocial interventions [45]. Notably, worrying causes patients to take positive or negative actions regarding the heart disease. At adequate levels, worrying is useful in promoting health-protective behaviours and adherence to treatment. However, high levels of stress can adversely affect health outcomes [46]. Furthermore, patients with high levels of anxiety have been reported to have fewer healthy coping strategies for stressful situations, an increased chance of adopting unhealthy behaviours, and non-adherence to treatment [47].

### Effect of biological and social characteristics to quality of life and health status

Similar to the findings of previous studies, we observed that women with CHD tended to have reduced QOL scores and increased risks of psychological problems compared to men [7, 34, 43, 48]. Indeed, differences in QOL and psychological problems between women and men were also found in the general Vietnamese population [31, 49] and other countries [50, 51]. This may be related to women’s tendencies to being sensitive to the social environment and worrying about the vulnerability to diseases [52].

Our study found significantly lower scores of QOL as measured by EQ-DS and EQ-VAS in older adults compared to younger adults. Certain research found lower QOL in older adults compared to the younger adults with CHD but others noted increased or unchanged QOL in older adults compared to younger adults [53]. A prior study found that older age plays a small role in predicting poor QOL, anxiety, and depression in adults with CHD [36]. In the current study, a logistic regression revealed that older age did not predict poor QOL and mental problems. Notably, we also found lower QOL scores in older patients compared to young patients than the general Vietnamese populations [31].

Multivariable logistic regression showed that the likelihood of depression in unmarried adults with CHD is greater compared to married adults with CHD and EQ-VAS in unmarried women was significantly lower compared to married women. With reference to previous studies, we noted that reduced QOL, anxiety, and depression were more common in unmarried than married individuals. This may be related to the disadvantages of the psychological distress and socioeconomic and psychosocial resources that appear frequently in unmarried compared to married individuals, especially in women [50, 54]. For example, married individuals often share financial expenses and receive sympathy from spouses, while unmarried individuals do not have such support.

In this study, educational level was associated with QOL and health status. Prevalence of poor QOL, anxiety, and depression in patients with less than a high school education was higher than patients with high school education and above. Although studies regarding educational level in adults with CHD in developing countries are limited, previous studies in developed countries have reported similar results between education level and QOL and mental disorders [7, 30, 48].

Our results also indicated that patients who had an unemployed status/unstable employment had a higher risk of having poor QOL, anxiety, and depression; this is consistent with the results of a previous study on adults with CHD [7]. Generally, unemployment or unstable employment negatively affect self-assessed health [55, 56]. We believe that the impact of unemployment or unstable employment on poor QOL results from declined financial consequences, reduced self-esteem, and barriers in social relationships. Therefore, patients with CHD should be provided with stable employment, which may help them cope with their illness, encourage them to overcome psychological barriers, and improve their social relationships and QOL.

Furthermore, we observed the negative effect of complex CHD/PAH on the QOL of patients with CHD. Previous studies have reported a negative association between regular physical activity and anxiety [57, 58]. Exercise and physical activity are useful to gain self-confidence. Exercise and physical activity are also good opportunities to meet or socialize with others and can help improve one’s mood and cope in a healthy way. However, exercise intolerance was common in patients with complex CHD or PAH [59].

The high rate of reduced QOL in our hospitalized adults with CHD was consistent with a recent study that showed the association between worse QOL and hospitalization in adults with CHD. Specifically, adults with CHD who had been hospitalized during the previous 12 months had significantly lower EQ-VAS and SWLS scores, and higher HADS-D scores than those who had not been hospitalized [60]. Notably, medical sources in developing countries are limited. For example, in our hospital—a national referral cardiovascular hospital— the number of specialists for adults with CHD was still not enough, especially in the outpatient clinic. This maybe because of inadequacies in diagnosis, treatment, and intervention and psychological problems in adults with CHD. In this context, we suggest a consistent approach such as a targeted screening programme for reduced QOL in hospitalized adults with CHD, which would be useful to understand QOL better. Then, we would have the information to adjust the appropriate health policies and screening programme for adults with CHD, including inpatients and outpatients, to improve their QOL.

To ensure the accuracy of patient self-reported information, a high number of inpatients with CHD were excluded in our study. We attempted to understand the reasons for the high number of inpatients who declined participation. Records during interviews of these patients noted that some patients could be classified as showing rejection of illness identity, consistent with adults with chronic disorders [61]. This suggested the need for a future study on illness identity in adults with CHD in Vietnam. Besides, we had given particular attention toward encouraging participants to fill up the questionnaire by themselves, which is useful in collecting the maximum number of accurate responses in patient self-reported outcome research.

### Limitations

This study had few limitations. First, this was a cross-sectional study, which does not prove causality between reduced QOL, health status, and biological and social characteristics. Second, although this study was conducted at a reference hospital in Vietnam, using a single location may have caused a sampling bias, which may have affected the representativeness of the study. Third, our study focused on hospitalized patients, which may present a higher rate of reduced QOL compared to outpatients. Fourth, exclusion of a high number of inpatients with CHD could cause biases. Fifth, using patient self-reported data may cause certain biases, since responses could be exaggerated or under-reported. Finally, because some subgroups had small samples, our study had just enough participants to divide each biological and social characteristic into two subgroups and analyse the differences.

## Conclusions

To the best of our knowledge, our study provides the first evidence on the high prevalence of poor QOL, anxiety, and depression in hospitalized adults with CHD in Vietnam. The findings highlight the relationship between poor QOL, life dissatisfaction, anxiety, depression, and biological-social characteristics including the female gender, older age, unmarried status, educational level lower than high school, unemployed status/unstable employment, and complex CHD/PAH. Therefore, we recommend that health-related QOL of hospitalized adults with CHD, especially for those presenting the abovementioned characteristics, should be screened and monitored.

## Supplementary Information

**Additional file 1.** Flow diagram of the selection strategy of adults with congenital heart disease.

**Additional file 2.** English version of EuroQOL-5 dimensions-5 level.

**Additional file 3.** English version of Satisfaction with Life Scale.

**Additional file 4.** English version of Hospital Anxiety and Depression Scale.

**Additional file 5.** The modified Vietnamese translation versions for quality of life and health status.

## Data Availability

The datasets used and/or analysed in the current study will be made available by the corresponding author upon reasonable request.

## Abbreviations

CHD: Congenital Heart Disease
CI: Confidence Intervals
EQ-5D-5L: EuroQOL-5 Dimensions-5 Level
EQ-DS: EuroQOL-Descriptive System
EQ-VAS: EuroQOL Visual Analogue Scale
HADS: Hospital Anxiety and Depression Scale
HADS-A: Hospital Anxiety and Depression Scale-Anxiety subscale
HADS-D: Hospital Anxiety and Depression Scale-Depression subscale
OR: Odds Ratios
PAH: Pulmonary Artery Hypertension
QOL: Quality of Life
SWLS: Satisfaction with Life Scale

## References

[1] Moons P, Bovijn L, Budts W, Belmans A, Gewillig M (2010). Temporal trends in survival to adulthood among patients born with congenital heart disease from 1970 to 1992 in Belgium. Circulation.

[2] Zimmerman MS, Smith AGC, Sable CA, Echko MM, Wilner LB, Olsen HE, Atalay HT, Awasthi A, Bhutta ZA, Boucher JL (2020). Global, regional, and national burden of congenital heart disease, 1990–2017: a systematic analysis for the Global Burden of Disease Study 2017. Lancet Child Adolescent Health.

[3] van der Linde D, Konings EE, Slager MA, Witsenburg M, Helbing WA, Takkenberg JJ, Roos-Hesselink JW (2011). Birth prevalence of congenital heart disease worldwide: a systematic review and meta-analysis. J Am Coll Cardiol.

[4] Fteropoulli T, Stygall J, Cullen S, Deanfield J, Newman SP (2013). Quality of life of adult congenital heart disease patients: a systematic review of the literature. Cardiol Young.

[5] Eslami B, Sundin O, Macassa G, Khankeh HR, Soares JJ (2013). Anxiety, depressive and somatic symptoms in adults with congenital heart disease. J Psychosom Res.

[6] Benderly M, Kalter-Leibovici O, Weitzman D, Blieden L, Buber J, Dadashev A, Mazor-Dray E, Lorber A, Nir A, Yalonetsky S (2019). Depression and anxiety are associated with high health care utilization and mortality among adults with congenital heart disease. Int J Cardiol.

[7] Vigl M, Niggemeyer E, Hager A, Schwedler G, Kropf S, Bauer U (2011). The importance of socio-demographic factors for the quality of life of adults with congenital heart disease. Qual Life Res.

[8] Park HK, Chun SY, Choi Y, Lee SY, Kim SJ, Park E-C (2015). Effects of social activity on health-related quality of life according to age and gender: an observational study. Health Qual Life Outcomes.

[9] Baumgartner H, De Backer J, Babu-Narayan SV, Budts W, Chessa M, Diller G-P, Lung B, Kluin J, Lang IM, Meijboom F, Moons P, Mulder BJM, Oechslin E, Roos-Hesselink JW, Schwerzmann M, Sondergaard L, Zeppenfeld K. ESC Scientific Document Group, 2020 ESC Guidelines for the management of adult congenital heart disease: The Task Force for the management of adult congenital heart disease of the European Society of Cardiology (ESC). Endorsed by: Association for European Paediatric and Congenital Cardiology (AEPC), International Society for Adult Congenital Heart Disease (ISACHD). Eur Heart J. 2021;42(6):563–645. 10.1093/eurheartj/ehaa554. 10.1093/eurheartj/ehaa554 32860028

[10] Apers S, Kovacs AH, Luyckx K, Thomet C, Budts W, Enomoto J, Sluman MA, Wang JK, Jackson JL, Khairy P (2016). Quality of life of adults with congenital heart disease in 15 countries: evaluating country-specific characteristics. J Am Coll Cardiol.

[11] Heusch A, Kahl HJ, Hensel KO, Calaminus G (2017). Health-related quality of life in paediatric patients with congenital heart defects: association with the type of heart defect and the surgical technique. Qual Life Res.

[12] Lawoko S, Soares JJF (2003). Quality of life among parents of children with congenital heart disease, parents of children with other diseases and parents of healthy children. Qual Life Res.

[13] Moons P, Luyckx K (2019). Quality-of-life research in adult patients with congenital heart disease: current status and the way forward. Acta Paediatr.

[14] Spijkerboer AW, Utens EMWJ, De Koning WB, Bogers AJJC, Helbing WA, Verhulst FC (2006). Health-related quality of life in children and adolescents after invasive treatment for congenital heart disease. Qual Life Res.

[15] Eslami B, Macassa G, Sundin Ö, Khankeh HR, Soares JJ (2015). Quality of life and life satisfaction among adults with and without congenital heart disease in a developing country. Eur J Prev Cardiol.

[16] Schrøder M, Boisen KA, Reimers J, Teilmann G, Brok J (2016). Quality of life in adolescents and young adults with CHD is not reduced: a systematic review and meta-analysis. Cardiol Young.

[17] Eaton SL, Wang Q, Menahem S (2017). Determinants of quality of life in adults with CHD: an Australian cohort. Cardiol Young.

[18] Apers S, Kovacs AH, Luyckx K, Alday L, Berghammer M, Budts W, Callus E, Caruana M, Chidambarathanu S, Cook SC (2015). Assessment of Patterns of Patient-Reported Outcomes in Adults with Congenital Heart disease—International Study (APPROACH-IS): rationale, design, and methods. Int J Cardiol.

[19] Phuc VM, Tin DN, Giang DTC (2015). Challenges in the management of congenital heart disease in Vietnam: a single center experience. Ann Pediatr Cardiol.

[20] Westhoff-Bleck M, Briest J, Fraccarollo D, Hilfiker-Kleiner D, Winter L, Maske U, Busch MA, Bleich S, Bauersachs J, Kahl KG (2016). Mental disorders in adults with congenital heart disease: unmet needs and impact on quality of life. J Affect Disord.

[21] Mai VQ, Sun S, Minh HV, Luo N, Giang KB, Lindholm L, Sahlen KG (2020). An EQ-5D-5L value set for Vietnam. Qual Life Res.

[22] Barton GR, Sach TH, Avery AJ, Jenkinson C, Doherty M, Whynes DK, Muir KR (2008). A comparison of the performance of the EQ-5D and SF-6D for individuals aged >or= 45 years. Health Econ.

[23] Diener E, Emmons RA, Larsen RJ, Griffin S (1985). The satisfaction with life scale. J Pers Assess.

[24] Zigmond AS, Snaith RP (1983). The hospital anxiety and depression scale. Acta Psychiatr Scand.

[25] Do TTH, Correa-Velez I, Dunne MP (2019). Trauma exposure and mental health problems among adults in central Vietnam: a randomized cross-sectional survey. Front Psychiatry.

[26] Truong DV, Bui QTT, Nguyen DT, Moore J (2019). Anxiety among inpatients with cancer: findings from a hospital-based cross-sectional study in Vietnam. Cancer Control.

[27] Marelli AJ, Mackie AS, Ionescu-Ittu R, Rahme E, Pilote L (2007). Congenital heart disease in the general population: changing prevalence and age distribution. Circulation.

[28] Wu MH, Lu CW, Chen HC, Kao FY, Huang SK (2018). Adult congenital heart disease in a nationwide population 2000–2014: epidemiological trends, arrhythmia, and standardized mortality ratio. J Am Heart Assoc.

[29] Xuan Tuan H, The Phuoc Long P, Duy Kien V, Manh Cuong L, Van Son N, Dalla-Pozza R (2019). Trends in the prevalence of atrial septal defect and its associated factors among congenital heart disease patients in Vietnam. J Cardiovasc Dev Dis.

[30] Pfitzer C, Helm PC, Rosenthal LM, Walker C, Ferentzi H, Bauer UMM, Berger F, Schmitt KRL (2018). Educational level and employment status in adults with congenital heart disease. Cardiol Young.

[31] Nguyen LH, Tran BX, Le Hoang QN, Tran TT, Latkin CA (2017). Quality of life profile of general Vietnamese population using EQ-5D-5L. Health Qual Life Outcomes.

[32] Ha NT, Duy HT, Le NH, Khanal V, Moorin R (2014). Quality of life among people living with hypertension in a rural Vietnam community. BMC Public Health.

[33] Tran BX, Moir MPI, Thai TPT, Nguyen LH, Ha GH, Nguyen THT, Truong NT, Latkin CA (2018). Socioeconomic inequalities in health-related quality of life among patients with cardiovascular diseases in Vietnam. Biomed Res Int.

[34] Berghammer M, Karlsson J, Ekman I, Eriksson P, Dellborg M (2013). Self-reported health status (EQ-5D) in adults with congenital heart disease. Int J Cardiol.

[35] Moons P, Apers S, Kovacs AH, Thomet C, Budts W, Enomoto J, Sluman MA, Wang JK, Jackson JL, Khairy P (2021). Sense of coherence in adults with congenital heart disease in 15 countries: Patient characteristics, cultural dimensions and quality of life. Eur J Cardiovasc Nurs J Work Group Cardiovasc Nurs European Soc Cardiol.

[36] Moons P, Kovacs AH, Luyckx K, Thomet C, Budts W, Enomoto J, Sluman MA, Yang HL, Jackson JL, Khairy P (2018). Patient-reported outcomes in adults with congenital heart disease: Inter-country variation, standard of living and healthcare system factors. Int J Cardiol.

[37] Tasiemski T, Priebe MM, Wilski M (2013). Life satisfaction and life values in people with spinal cord injury living in three Asian countries: a multicultural study. J Spinal Cord Med.

[38] Oishi S, Diener EF, Lucas RE, Suh EM (1999). Cross-cultural variations in predictors of life satisfaction: perspectives from needs and values. Pers Soc Psychol Bull.

[39] Jagodzinski W (2010). Economic, social, and cultural determinants of life satisfaction: are there differences between Asia and Europe?. Soc Indic Res.

[40] Vuong DA, Van Ginneken E, Morris J, Ha ST, Busse R (2011). Mental health in Vietnam: Burden of disease and availability of services. Asian J Psychiatr.

[41] Eslami B (2017). Correlates of posttraumatic stress disorder in adults with congenital heart disease. Congenit Heart Dis.

[42] Jackson JL, Misiti B, Bridge JA, Daniels CJ, Vannatta K (2015). Emotional functioning of adolescents and adults with congenital heart disease: a meta-analysis. Congenit Heart Dis.

[43] Kovacs AH, Saidi AS, Kuhl EA, Sears SF, Silversides C, Harrison JL, Ong L, Colman J, Oechslin E, Nolan RP (2009). Depression and anxiety in adult congenital heart disease: predictors and prevalence. Int J Cardiol.

[44] Bang JS, Jo S, Kim GB, Kwon BS, Bae EJ, Noh CI, Choi JY (2013). The mental health and quality of life of adult patients with congenital heart disease. Int J Cardiol.

[45] Diller G-P, Bräutigam A, Kempny A, Uebing A, Alonso-Gonzalez R, Swan L, Babu-Narayan SV, Baumgartner H, Dimopoulos K, Gatzoulis MA (2015). Depression requiring anti-depressant drug therapy in adult congenital heart disease: prevalence, risk factors, and prognostic value. Eur Heart J.

[46] Bremner JD, Campanella C, Khan Z, Shah M, Hammadah M, Wilmot K, Al Mheid I, Lima BB, Garcia EV, Nye J (2018). Brain correlates of mental stress-induced Myocardial Ischemia. Psychosom Med.

[47] Abed MA, Kloub MI, Moser DK (2014). Anxiety and adverse health outcomes among cardiac patients: a biobehavioral model. J Cardiovasc Nurs.

[48] Chen CA, Liao SC, Wang JK, Chang CI, Chiu IS, Chen YS, Lu CW, Lin MT, Chiu HH, Chiu SN (2011). Quality of life in adults with congenital heart disease: biopsychosocial determinants and sex-related differences. Heart (British Cardiac Society).

[49] Nguyen T, Tran T, Tran H, Tran T, Fisher J (2019). The burden of clinically significant symptoms of common and severe mental disorders among adults in Vietnam: a population-based cross-sectional survey. BMC Public Health.

[50] Han KT, Park EC, Kim JH, Kim SJ, Park S (2014). Is marital status associated with quality of life?. Health Qual Life Outcomes.

[51] Campos ACV (2014). e Ferreira EF, Vargas AMD, Albala C: Aging, Gender and Quality of Life (AGEQOL) study: factors associated with good quality of life in older Brazilian community-dwelling adults. Health Qual Life Outcomes.

[52] McLean CP, Asnaani A, Litz BT, Hofmann SG (2011). Gender differences in anxiety disorders: prevalence, course of illness, comorbidity and burden of illness. J Psychiatr Res.

[53] Apers S, Luyckx K, Moons P (2013). Quality of life in adult congenital heart disease: what do we already know and what do we still need to know?. Curr Cardiol Rep.

[54] Bierman A, Fazio EM, Milkie MA (2006). A multifaceted approach to the mental health advantage of the married: assessing how explanations vary by outcome measure and unmarried group. J Fam Issues.

[55] McKee-Ryan F, Song Z, Wanberg CR, Kinicki AJ (2005). Psychological and physical well-being during unemployment: a meta-analytic study. J Appl Psychol.

[56] Norström F, Virtanen P, Hammarström A, Gustafsson PE, Janlert U (2014). How does unemployment affect self-assessed health? A systematic review focusing on subgroup effects. BMC Public Health.

[57] Sareen J, Cox BJ, Clara I, Asmundson GJ (2005). The relationship between anxiety disorders and physical disorders in the U.S. National Comorbidity Survey. Depress Anxiety.

[58] Goodwin RD (2003). Association between physical activity and mental disorders among adults in the United States. Prev Med.

[59] Diller G-P, Dimopoulos K, Okonko D, Li W, Babu-Narayan SV, Broberg CS, Johansson B, Bouzas B, Mullen MJ, Poole-Wilson PA (2005). Exercise intolerance in adult congenital heart disease. Circulation.

[60] Moons P, Luyckx K, Thomet C, Budts W, Enomoto J, Sluman MA, Wang J-K, Jackson JL, Khairy P, Cook SC, et al. Patient-reported outcomes in adults with congenital heart disease following hospitalization (from APPROACH-IS). Am J Cardiol. 2021;145:135–142. 10.1016/j.amjcard.2020.12.088. 10.1016/j.amjcard.2020.12.088 33460605

[61] Oris L, Luyckx K, Rassart J, Goubert L, Goossens E, Apers S, Arat S, Vandenberghe J, Westhovens R, Moons P (2018). Illness identity in adults with a chronic illness. J Clin Psychol Med Settings.

