# Supplementary material for: Quality of life and health status of hospitalized adults with congenital heart disease in Vietnam: a cross-sectional study
Source: BMC Cardiovasc Disord. 2021 May 5;21:229. doi: 10.1186/s12872-021-02026-1 (PMC8097946; doi:10.1186/s12872-021-02026-1)
Supplement: Supplementary file 1 — Additional file 1. Flow diagram of the selection strategy of adults with congenital heart disease. [file 12872_2021_2026_MOESM1_ESM.docx]

Quality of life and health status of hospitalized adults with congenital heart disease in Vietnam: A cross-sectional study

Thanh-Huong Truong, Ngoc-Thanh Kim, Mai-Ngoc Thi Nguyen, Doan-Loi Do, Hong Thi Nguyen, Thanh-Tung Le, Hong-An Le

**Supplementary 1 Flow diagram of the selection strategy of adults with congenital heart disease**


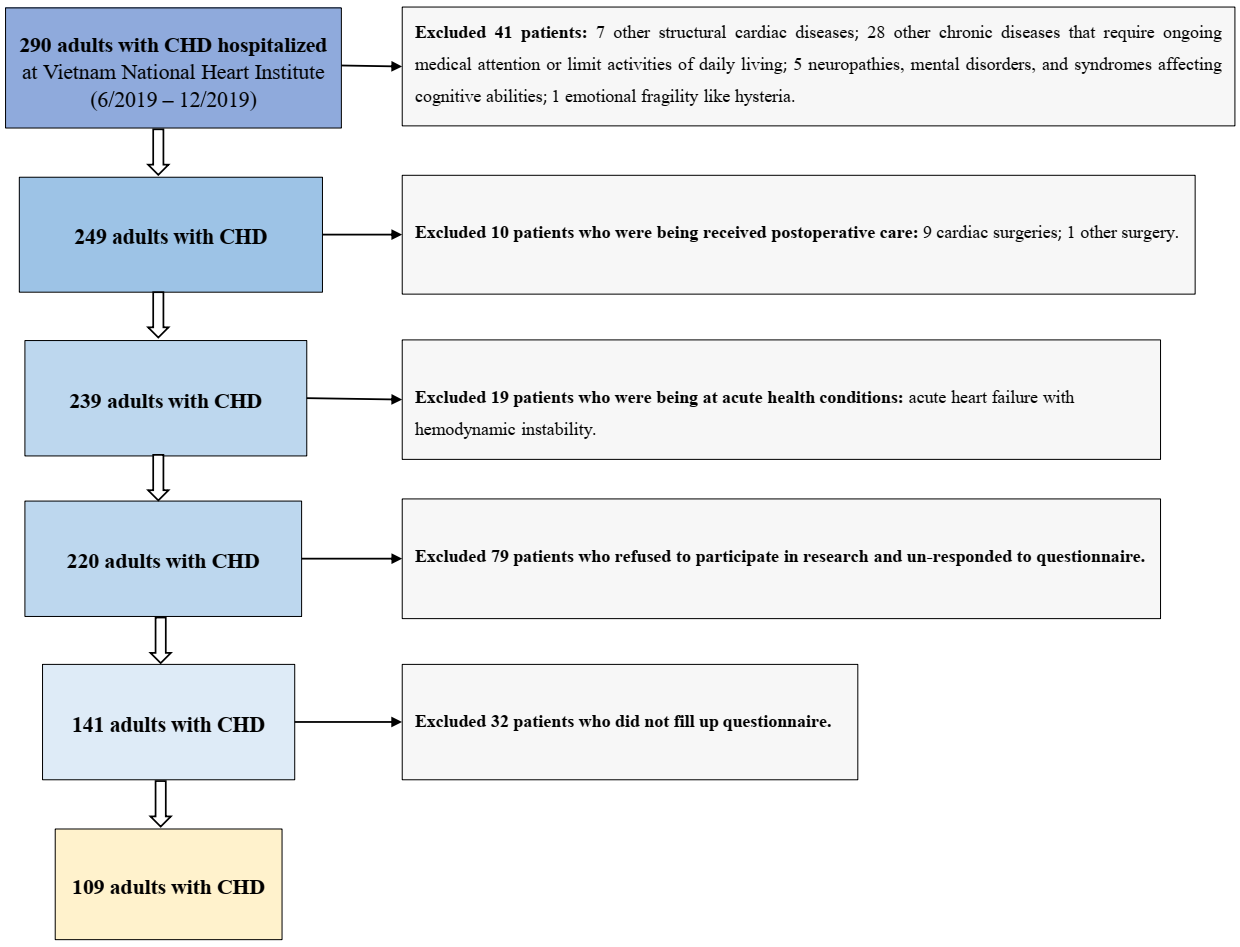


CHD: Congenital heart disease
