# Supplementary material for: Quality of life and health status of hospitalized adults with congenital heart disease in Vietnam: a cross-sectional study
Source: BMC Cardiovasc Disord. 2021 May 5;21:229. doi: 10.1186/s12872-021-02026-1 (PMC8097946; doi:10.1186/s12872-021-02026-1)

Quality of life and health status of hospitalized adults with congenital heart disease in Vietnam: A cross-sectional study

Thanh-Huong Truong, Ngoc-Thanh Kim, Mai-Ngoc Thi Nguyen, Doan-Loi Do, Hong Thi Nguyen, Thanh-Tung Le, Hong-An Le

**Supplementary 2 English version of EuroQOL-5 dimensions-5 level**

<https://euroqol.org/eq-5d-instruments/sample-demo/>

***EQ-Descriptive System***

*Under each heading, please tick the ONE box that best describes your health TODAY.*

# MOBILITY

I have no problems in walking about 

I have slight problems in walking about 

I have moderate problems in walking about 

I have severe problems in walking about 

I am unable to walk about 

# SELF-CARE

I have no problems washing or dressing myself 

I have slight problems washing or dressing myself 

I have moderate problems washing or dressing myself 

I have severe problems washing or dressing myself 

I am unable to wash or dress myself 

USUAL ACTIVITIES *(e.g. work, study, housework, family or leisure activities)*

I have no problems doing my usual activities 

I have slight problems doing my usual activities 

I have moderate problems doing my usual activities 

I have severe problems doing my usual activities 

I am unable to do my usual activities 

# PAIN / DISCOMFORT

I have no pain or discomfort 

I have slight pain or discomfort 

I have moderate pain or discomfort 

I have severe pain or discomfort 

I have extreme pain or discomfort 

# ANXIETY / DEPRESSION

I am not anxious or depressed 

I am slightly anxious or depressed 

I am moderately anxious or depressed 

I am severely anxious or depressed 

I am extremely anxious or depressed

***EQ-Visual Analogue Scale***

- We would like to know how good or bad your health is TODAY.
- This scale is numbered from 0 to 100.
- 100 means the best health you can imagine.
- 0 means the worst health you can imagine.
- Mark an X on the scale to indicate how your health is TODAY.
- Now, please write the number you marked on the scale in the box


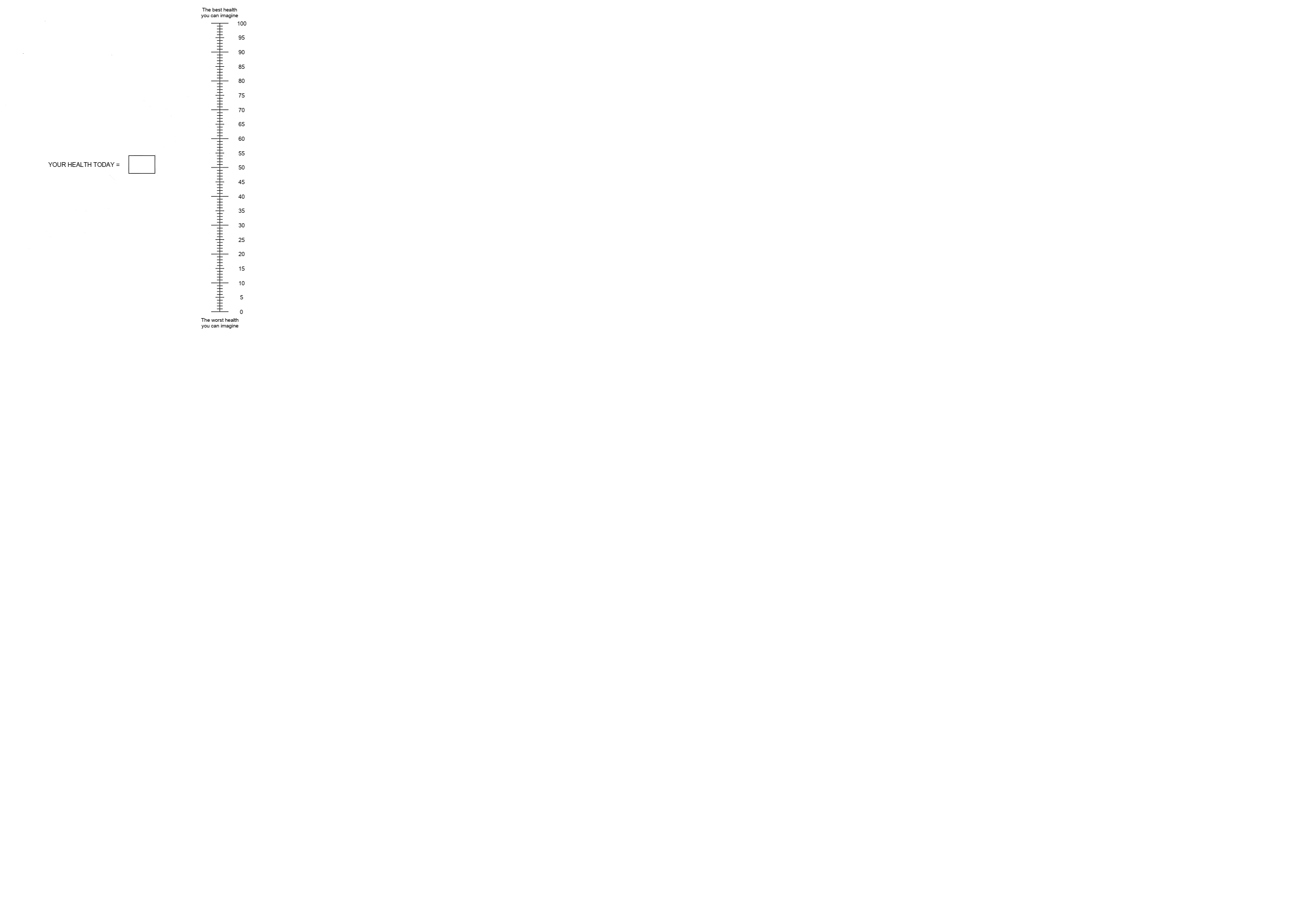

Supplement: Supplementary file 2 — Additional file 2. English version of EuroQOL-5 dimensions-5 level. [file 12872_2021_2026_MOESM2_ESM.docx]
