# Supplementary material for: Quality of life and health status of hospitalized adults with congenital heart disease in Vietnam: a cross-sectional study
Source: BMC Cardiovasc Disord. 2021 May 5;21:229. doi: 10.1186/s12872-021-02026-1 (PMC8097946; doi:10.1186/s12872-021-02026-1)
Supplement: Supplementary file 3 — Additional file 3. English version of Satisfaction with Life Scale. [file 12872_2021_2026_MOESM3_ESM.docx]

Quality of life and health status of hospitalized adults with congenital heart disease in Vietnam: A cross-sectional study

Thanh-Huong Truong, Ngoc-Thanh Kim, Mai-Ngoc Thi Nguyen, Doan-Loi Do, Hong Thi Nguyen, Thanh-Tung Le, Hong-An Le

**Supplementary 3 English version of Satisfaction with Life Scale**

<http://labs.psychology.illinois.edu/~ediener/SWLS.html>

Below are five statements that you may agree or disagree with. Using the 1 - 7 scale below, indicate your agreement with each item by placing the appropriate number on the line preceding that item. Please be open and honest in your responding.

- 7 - Strongly agree
- 6 - Agree
- 5 - Slightly agree
- 4 - Neither agree nor disagree
- 3 - Slightly disagree
- 2 - Disagree
- 1 - Strongly disagree

____ In most ways my life is close to my ideal.

____ The conditions of my life are excellent.

____ I am satisfied with my life.

____ So far I have gotten the important things I want in life.

____ If I could live my life over, I would change almost nothing.

- - - 31 - 35 Extremely satisfied
    - 26 - 30 Satisfied
    - 21 - 25 Slightly satisfied
    - 20        Neutral
    - 15 - 19 Slightly dissatisfied
    - 10 - 14 Dissatisfied
    - 5 -  9   Extremely dissatisfied
