# Supplementary material for: Quality of life and health status of hospitalized adults with congenital heart disease in Vietnam: a cross-sectional study
Source: BMC Cardiovasc Disord. 2021 May 5;21:229. doi: 10.1186/s12872-021-02026-1 (PMC8097946; doi:10.1186/s12872-021-02026-1)
Supplement: Supplementary file 4 — Additional file 4. English version of Hospital Anxiety and Depression Scale. [file 12872_2021_2026_MOESM4_ESM.docx]

Quality of life and health status of hospitalized adults with congenital heart disease in Vietnam: A cross-sectional study

Thanh-Huong Truong, Ngoc-Thanh Kim, Mai-Ngoc Thi Nguyen, Doan-Loi Do, Hong Thi Nguyen, Thanh-Tung Le, Hong-An Le

Supplementary 4 English version of Hospital Anxiety and Depression Scale

<https://www.svri.org/sites/default/files/attachments/2016-01-13/HADS.pdf>

**Tick the box beside the reply that is closest to how you have been feeling in the past week.**

**Don’t take too long over you replies: your immediate is best.**

| **D** | **A** |  | **D** | **A** |  |
| --- | --- | --- | --- | --- | --- |
|  |  | **I feel tense or 'wound up':** |  |  | **I feel as if I am slowed down:** |
|  | 3 | Most of the time | 3 |  | Nearly all the time |
|  | 2 | A lot of the time | 2 |  | Very often |
|  | 1 | From time to time, occasionally | 1 |  | Sometimes |
|  | 0 | Not at all | 0 |  | Not at all |
|  |  |  |  |  |  |
|  |  | **I still enjoy the things I used to enjoy:** |  |  | **I get a sort of frightened feeling like 'butterflies' in the stomach:** |
| 0 |  | Definitely as much |  | 0 | Not at all |
| 1 |  | Not quite so much |  | 1 | Occasionally |
| 2 |  | Only a little |  | 2 | Quite Often |
| 3 |  | Hardly at all |  | 3 | Very Often |
|  |  |  |  |  |  |
|  |  | **I get a sort of frightened feeling as if something awful is about to happen:** |  |  | **I have lost interest in my appearance:** |
|  | 3 | Very definitely and quite badly | 3 |  | Definitely |
|  | 2 | Yes, but not too badly | 2 |  | I don't take as much care as I should |
|  | 1 | A little, but it doesn't worry me | 1 |  | I may not take quite as much care |
|  | 0 | Not at all | 0 |  | I take just as much care as ever |
|  |  |  |  |  |  |
|  |  | **I can laugh and see the funny side of things:** |  |  | **I feel restless as I have to be on the move:** |
| 0 |  | As much as I always could |  | 3 | Very much indeed |
| 1 |  | Not quite so much now |  | 2 | Quite a lot |
| 2 |  | Definitely not so much now |  | 1 | Not very much |
| 3 |  | Not at all |  | 0 | Not at all |
|  |  | **Worrying thoughts go through my mind:** |  |  | **I look forward with enjoyment to things:** |
|  | 3 | A great deal of the time | 0 |  | As much as I ever did |
|  | 2 | A lot of the time | 1 |  | Rather less than I used to |
|  | 1 | From time to time, but not too often | 2 |  | Definitely less than I used to |
|  | 0 | Only occasionally | 3 |  | Hardly at all |
|  |  |  |  |  |  |
|  |  | **I feel cheerful:** |  |  | **I get sudden feelings of panic:** |
| 3 |  | Not at all |  | 3 | Very often indeed |
| 2 |  | Not often |  | 2 | Quite often |
| 1 |  | Sometimes |  | 1 | Not very often |
| 0 |  | Most of the time |  | 0 | Not at all |
|  |  |  |  |  |  |
|  |  | **I can sit at ease and feel relaxed:** |  |  | **I can enjoy a good book or radio or TV program:** |
|  | 0 | Definitely | 0 |  | Often |
|  | 1 | Usually | 1 |  | Sometimes |
|  | 2 | Not Often | 2 |  | Not often |
|  | 3 | Not at all | 3 |  | Very seldom |

Please check you have answered all the questions Scoring:

Total score: Depression (D) Anxiety (A)

0-7 = Normal

8-10 = Borderline abnormal (borderline case)

11-21 = Abnormal (case)
