# Supplementary material for: Quality of life and health status of hospitalized adults with congenital heart disease in Vietnam: a cross-sectional study
Source: BMC Cardiovasc Disord. 2021 May 5;21:229. doi: 10.1186/s12872-021-02026-1 (PMC8097946; doi:10.1186/s12872-021-02026-1)
Supplement: Supplementary file 5 — Additional file 5. The modified Vietnamese translation versions for quality of life and health status. [file 12872_2021_2026_MOESM5_ESM.docx]

Quality of life and health status of hospitalized adults with congenital heart disease in Vietnam: A cross-sectional study

Thanh-Huong Truong, Ngoc-Thanh Kim, Mai-Ngoc Thi Nguyen, Doan-Loi Do, Hong Thi Nguyen, Thanh-Tung Le, Hong-An Le

**Supplementary 5 The modified Vietnamese translation versions for quality of life and health status**

**A. The modified Vietnamese translation version of the EQ-5D-5L**

***The modified Vietnamese translation version of the EQ-DS***

ĐI LẠI

🞎 Tôi đi lại không khó khăn

🞎 Tôi đi lại hơi khó khăn

🞎 Tôi đi lại khá khó khăn

🞎 Tôi đi lại rất khó khăn

🞎 Tôi không thể đi lại được

TỰ CHĂM SÓC

🞎 Tôi thấy không khó khăn gì khi tự tắm rửa hay khi tự mặc quần áo

🞎 Tôi thấy hơi khó khăn khi tự tắm rửa hay khi tự mặc quần áo

🞎 Tôi thấy khá khó khăn khi tự tắm rửa hay khi tự mặc quần áo

🞎 Tôi thấy rất khó khăn khi tự tắm rửa hay khi tự mặc quần áo

🞎 Tôi không thể tự tắm rửa hay không thể tự mặc quần áo

SINH HOẠT THƯỜNG LỆ (ví dụ: làm việc, học hành, làm việc nhà các hoạt động trong gia đình, vui chơi giải trí)

🞎 Tôi thấy không khó khăn gì khi thực hiện các sinh hoạt thường lệ của tôi

🞎 Tôi thấy hơi khó khăn khi thực hiện các sinh hoạt thường lệ của tôi

🞎 Tôi thấy khá khó khăn khi thực hiện các sinh hoạt thường lệ của tôi

🞎 Tôi thấy rất khó khăn khi thực hiện các sinh hoạt thường lệ của tôi

🞎 Tôi không thể thực hiện các sinh hoạt thường lệ của tôi

ĐAU/ KHÓ CHỊU

🞎 Tôi không đau hay không khó chịu

🞎 Tôi hơi đau hay hơi khó chịu

🞎 Tôi khá đau hay khá khó chịu

🞎 Tôi rất đau hay rất khó chịu

🞎 Tôi cực kỳ đau hay cực kỳ khó chịu

LO LẮNG/ U SẦU

🞎 Tôi không lo lắng hay không u sầu

🞎 Tôi thấy hơi lo lắng hay hơi u sầu một chút

🞎 Tôi thấy khá lo lắng hay khá u sầu

🞎 Tôi thấy rất lo lắng hay rất u sầu

🞎 Tôi thấy cực kỳ lo lắng hay cực kỳ u sầu

***The modified Vietnamese translation version of the EQ-VAS***

| Nhằm giúp mọi người có thể xác định trạng thái tốt hay xấu của sức khỏe, chúng tôi vẽ ra một thang điểm (giống như nhiệt kế). Ở thang điểm này, số điểm 100 tương ứng với tình trạng sức khỏe tốt nhất và 0 tương ứng với tình trạng sức khỏe xấu nhất mà anh/chị có thể hình dung được. |
| --- |
| Chúng tôi mong muốn anh/chị sẽ chỉ ra trên thang điểm này tình trạng sức khỏe (tốt hay xấu) của mình ngày hôm nay.  ***Xin hãy vẽ 1 đường kéo ngang từ ô bên dưới đến mức điểm mà anh/chị cho là thích ứng nhất với tình trạng sức khỏe của mình hôm nay.*** |

Tình trạng sức khỏe tốt nhất có thể hình dung được

9 0

8 0

7 0

6 0

5 0

4 0

3 0

2 0

1 0

100

0

Điểm tình trạng sức khỏe (tốt hay xấu) của anh/chị ngày
hôm nay

Tình trạng sức khỏe xấu nhất có thể hình dung được

**B. The modified Vietnamese translation version of the SWLS**

*Xin anh/chị cho biết mức độ hài lòng với cuộc sống của bản thân với 5 đề mục sau.*

**1. Hầu hết những điều tôi đã trải qua gần như đều là những điều mà tôi dự định trước đó.**

🞎 Tôi đồng ý hoàn toàn với điều này

🞎 Tôi gần như đồng ý với điều này

🞎 Tôi đồng ý một phần với điều này

🞎 Tôi phân vân giữa đồng ý hay không đồng ý với điều này

🞎 Tôi không đồng ý một phần với điều này

🞎 Tôi gần như không đồng ý với điều này

🞎 Tôi không đồng ý hoàn toàn với điều này

**2. Điều kiện cuộc sống hiện tại của tôi thật tuyệt vời.**

🞎 Tôi đồng ý hoàn toàn với điều này

🞎 Tôi gần như đồng ý với điều này

🞎 Tôi đồng ý một phần với điều này

🞎 Tôi phân vân giữa đồng ý hay không đồng ý với điều này

🞎 Tôi không đồng ý một phần với điều này

🞎 Tôi gần như không đồng ý với điều này

🞎 Tôi không đồng ý hoàn toàn với điều này

**3. Tôi hài lòng với cuộc sống hiện tại.**

🞎 Tôi đồng ý hoàn toàn với điều này

🞎 Tôi gần như đồng ý với điều này

🞎 Tôi đồng ý một phần với điều này

🞎 Tôi phân vân giữa đồng ý hay không đồng ý với điều này

🞎 Tôi không đồng ý một phần với điều này

🞎 Tôi gần như không đồng ý với điều này

🞎 Tôi không đồng ý hoàn toàn với điều này

**4. Cho đến hiện tại, tôi đã đạt được những điều cần thiết mà tôi mong muốn.**

🞎 Tôi đồng ý hoàn toàn với điều này

🞎 Tôi gần như đồng ý với điều này

🞎 Tôi đồng ý một phần với điều này

🞎 Tôi phân vân giữa đồng ý hay không đồng ý với điều này

🞎 Tôi không đồng ý một phần với điều này

🞎 Tôi gần như không đồng ý với điều này

🞎 Tôi không đồng ý hoàn toàn với điều này

**5. Nếu được sống lại từ đầu, tôi vẫn muốn sống cuộc sống như hiện tại.**

🞎 Tôi đồng ý hoàn toàn với điều này

🞎 Tôi gần như đồng ý với điều này

🞎 Tôi đồng ý một phần với điều này

🞎 Tôi phân vân giữa đồng ý hay không đồng ý với điều này

🞎 Tôi không đồng ý một phần với điều này

🞎 Tôi gần như không đồng ý với điều này

🞎 Tôi không đồng ý hoàn toàn với điều này

**C. The modified Vietnamese translation version of the HADS**

*Anh chị hãy cho chúng tôi biết câu trả lời gần nhất với cảm giác của anh chị trong tuần qua. Không cần mất quá nhiều thời gian để đưa ra câu trả lời. Chọn câu trả lời xuất hiện đầu tiên trong suy nghĩ của anh chị.*

**1. Anh chị cảm thấy căng thẳng**

🞎 Hầu như lúc nào cũng có cảm giác này

🞎 Rất hay có cảm giác này

🞎 Thỉnh thoảng có cảm giác này

🞎 Hầu như không có cảm giác này

**2. Anh chị cảm thấy bản thân tâm trạng đang xấu đi**

🞎 Lúc nào cũng thấy vậy

🞎 Rất thường xuyên

🞎 Thỉnh thoảng

🞎 Hầu như không

**3. Anh chị vẫn có cảm giác thích thú với những sở thích trước đây**

🞎 Chắc chắn là rất nhiều

🞎 Không quá nhiều

🞎 Chỉ một chút

🞎 Hầu như là không

**4. Anh chị cảm thấy bồn chồn, lo lắng**

🞎 Chưa từng

🞎 Thỉnh thoảng

🞎 Rất thường xuyên

🞎 Cực kỳ thường xuyên

**5. Anh chị cảm thấy sợ hãi như thể có điều gì không hay sắp xảy ra**

🞎 Điều này có với dự cảm điều đó rất tồi tệ

🞎 Điều này có và dự cảm điều này không quá tồi tệ

🞎 Có cảm giác một chút nhưng sự sợ hãi này không đáng kể

🞎 Không có cảm giác đó

**6. Anh chị chẳng còn quan tâm tới dáng vẻ bản thân**

🞎 Đúng vậy, tôi chẳng còn quan tâm tới dáng vẻ bản thân ra sao nữa

🞎 Tôi không quan tâm quá nhiều tới dáng vẻ bản thân

🞎 Tôi có lẽ không quá quan tâm đến dáng vẻ bản thân

🞎 Tôi vẫn quan tâm rất nhiều như bình thường

**7. Anh chị vẫn lạc quan với mọi việc xung quanh**

🞎 Rất nhiều

🞎 Còn nhiều nhưng có giảm đi

🞎 Không nhiều lắm

🞎 Hầu như không

**8. Anh chị có cảm thấy bồn chồn mỗi khi phải đi đâu đó**

🞎 Hầu như lúc nào cũng bồn chồn khi phải đi đâu đó

🞎 Rất nhiều lần có cảm giác bồn chồn khi phải đi đâu đó

🞎 Chỉ đôi khi có cảm giác bồn chồn khi phải đi đâu đó

🞎 Hầu như không có cảm giác này

**9. Những suy nghĩ lo lắng xuất hiện trong đầu anh chị ra sao**

🞎 Gần như lúc nào cũng vậy

🞎 Rất nhiều lần

🞎 Chỉ xuất hiện đôi lúc chứ không quá nhiều

🞎 Thỉnh thoảng

**10. Anh chị luôn mong đợi tận hưởng nhiều thứ**

🞎 Rất nhiều

🞎 Ít khi

🞎 Rất là ít

🞎 Hầu như không

**11. Anh chị cảm thấy vui vẻ**

🞎 Hầu như không

🞎 Không thường xuyên

🞎 Chỉ thỉnh thoảng

🞎 Gần như mọi lúc

**12. Anh chị đột nhiên cảm thấy hoảng loạn**

🞎 Rất nhiều lần

🞎 Nhiều lần

🞎 Không quá thường xuyên

🞎 Hầu như không

**13. Anh chị có thể ngồi thoải mái và cảm thấy thư giãn**

🞎 Rất thường xuyên

🞎 Thường xuyên

🞎 Không thường xuyên

🞎 Hầu như không

**14. Anh chị có cảm giác hứng thú đọc sách hoặc nghe chương trình đài hoặc xem chương trình tivi**

🞎 Thường xuyên

🞎 Thỉnh thoảng

🞎 Không thường xuyên

🞎 Rất hiếm khi
